# Schistosome esophageal gland factor MEG-8.2 drives host cell lysis and interacts with host immune proteins

**DOI:** 10.1371/journal.ppat.1014044

**Published:** 2026-03-11

**Authors:** Pallavi Yadav, Sabona B. Simbassa, Ryan Sloan, Phillip A. Newmark, Jayhun Lee

**Affiliations:** 1 Department of Microbiology and Molecular Genetics, McGovern Medical School, The University of Texas Health Science Center at Houston, Houston, Texas, United States of America; 2 Microbiology and Infectious Diseases Program, The University of Texas MD Anderson Cancer Center UTHealth Houston Graduate School of Biomedical Sciences, Houston, Texas, United States of America; 3 Howard Hughes Medical Institute, Morgridge Institute for Research, Department of Integrative Biology, University of Wisconsin–Madison, Madison, Wisconsin, United States of America; University of Utah, UNITED STATES OF AMERICA

## Abstract

Schistosomes are blood flukes that ingest large amounts of host blood during their intra-mammalian stage. The ingested blood contains leukocytes that can be harmful, yet the parasites survive inside the host for decades, reflecting superb immune evasion mechanisms that remain poorly understood. Our previous work discovered that FoxA, a forkhead transcription factor, drives the production of the esophageal gland, an anterior digestive organ essential for degrading ingested leukocytes and for *in vivo* survival. However, a comprehensive molecular makeup of the esophageal gland remains unclear. Importantly, the esophageal gland factors responsible for degrading ingested leukocytes, their mechanisms of action, and how such a function relates to parasite survival and immune evasion remain unknown. Here, we identify additional esophageal gland genes by taking a comparative transcriptomics approach to identify transcripts altered in *foxA* knockdown adult schistosomes. A targeted RNAi screen coupled with biochemistry reveals that specific domains of the micro-exon gene MEG-8.2, can drive host cell lysis in a concentration-dependent manner. Using pull-down assays coupled with mass spectrometry, we discover that MEG-8.2 interacts with several host membrane and extracellular proteins that play important roles in activating innate and/or adaptive immunity. Together, our findings suggest a dual role for MEG-8.2 in effectively lysing the ingested cells in the esophageal lumen and interacting with specific host proteins to neutralize or suppress host immunity. These findings lay an important foundation for exploiting esophageal gland factors to treat schistosomiasis.

## Author summary

Schistosomiasis is a major parasitic disease that affects over 200 million people. Schistosomes’ ability to thrive inside the hostile environment of the host bloodstream and reproduce is the root cause of the disease. One potential source of such an ability comes from an elusive mechanism that neutralizes harmful immune components ingested during blood feeding. Previously, we discovered that a forkhead transcription factor, FoxA, drives the production of the esophageal gland, an anterior digestive organ essential for degrading ingested host leukocytes and for *in vivo* parasite survival. Here, we use *foxA* RNAi parasites to comprehensively identify esophageal gland factors. Using a targeted RNAi screen coupled with biochemistry, we discover that a specific domain of MEG-8.2, a micro-exon gene family protein, is necessary and sufficient to directly lyse host blood cells in a concentration-dependent manner. Furthermore, using pull-down mass spectrometry and protein modeling, we find that specific MEG-8.2 domains play an essential role in interacting with specific host proteins with known roles in immune activation. Our findings propose a dual role for MEG-8.2: concentration-dependent lysis of the host blood cell membrane and interaction with host proteins essential for the proper functioning of immune defense.

## Introduction

During intramammalian homeostasis, adult schistosomes reproduce within the host vasculature, producing hundreds of eggs daily, which causes schistosomiasis [1]. Schistosomiasis remains one of the most prevalent parasitic diseases, with over 200 million individuals affected globally [2]. However, other than praziquantel, no treatment or preventative options are available, highlighting the urgent need to devise alternative approaches to target these parasites. Contrary to schistosome eggs, which are highly immunogenic, worms inside the host bloodstream can withstand attacks by the immune system, leading to their longevity. Previous studies have revealed the importance of the tegument, a syncytial outer skin of the parasite, to play a crucial role in this process. The tegument is structurally unique: the parasite-host interface is composed of a double lipid bilayer, a feature found uniquely among blood flukes [3]. In addition, host glycolipids and glycoproteins are found on the tegument, suggesting that their acquisition might help the parasite avoid detection by the host immune system [4]. More recently, parasite stem cells have emerged as the prime suspect in the successful development, homeostasis, reproduction, and propagation across the life cycle [5,6]. In adults, a significant proportion of somatic stem cells differentiate into tegument cells via transcription factors such as Zfp-1–1, p53-1, and Klf4 to replenish the tegument, which undergoes high cellular turnover [7–10]. Similar mechanisms have been reported for tissues underlying parasites’ digestive tract, in which transcription factor Hnf4 (hepatocyte nuclear factor 4) plays a crucial role in gut cell production and maintenance [11]. Hnf4 knockdown parasites display increased *eled*+ (stem/progenitor) cells and decreased output of *ctsb*+ gut cells, perturbing the digestion of red blood cells, resulting in parasite death *in vivo*.

Given that schistosomes consume large amounts of host blood containing potentially harmful immune components, these parasites likely deploy immune-evasion mechanisms through their digestive tract. Anterior to the gut is the parasites’ esophagus, which is surrounded by a digestive organ called the esophageal gland (EG). The EG has been suggested as the initial site of blood processing, where ingested erythrocytes are broken down and damaged leukocytes appear tethered to the esophageal lumen [12]. There are ~ 1000 cells in an adult male EG [12]. These cells are densely packed, and their cytoplasm extends into the esophageal lumen for secretion. Electron microscopy shows secretory granules and vesicles found throughout the cytoplasm. These cytoplasmic extensions form a 2-dimensional plate-like structure that runs longitudinally from anterior to posterior of the lumen [12]. These plates are regularly spaced, creating a large luminal surface area for processing ingested blood. These observations suggest that the cell types and genes that comprise the EG orchestrate secretion and maintain tissue integrity and function.

Our recent work discovered an essential regulator of EG cell development and maintenance, a forkhead transcription factor, FoxA [13]. Expression of *foxA* is enriched in the EG and its neighboring *h2b*+ stem/progenitor cells. Knockdown of *foxA* in adult schistosomes disrupted the expression of several EG genes, preventing the parasites from blocking and degrading leukocytes in the esophagus. Such a function appears essential for evading the host immune system: parasites lacking the EG are rapidly cleared from the bloodstream of immunocompetent mice, while they can survive inside immunocompromised mice. Previous studies identified several genes enriched in the EG. For instance, several EG genes were discovered by comparing the transcriptomes obtained from anterior and posterior halves of adult parasites and validating the expression of anterior-enriched genes using whole-mount mRNA *in situ* hybridization (WISH) [14]. More recently, single-cell RNA-sequencing (scRNA-seq) of parasites from different life cycle stages captured a few dozen EG cells with a handful of genes [11]. However, many of these have not been validated as *bona fide* EG genes. Importantly, their functional role in blocking or degrading the ingested blood cells and their role in parasite survival and parasite-host interaction remains to be determined. In this study, we use *foxA* RNAi RNA-seq coupled with *in situ* hybridization to comprehensively identify EG genes. We use RNAi to systematically screen for candidate EG factors involved in degrading the ingested leukocytes. Furthermore, we employ truncation mutagenesis, cytotoxicity assay, pull-down, mass spectrometry, and *in silico* protein interaction modeling to dissect the functional domains of the top candidate factors that disrupt the host cell membrane and interact with a specific set of host proteins. Our findings lead us to propose a concentration-dependent dual role of the identified EG factor in degrading the host cells and interacting with host cell proteins with known functions in immune-mediated defense against pathogens. Together, this study establishes the foundation for deciphering the mechanism of action of EG-released factors and raises the possibility of targeting these factors as therapeutic avenues for treating schistosomiasis.

## Results

### *foxA* RNAi RNA-seq comprehensively identifies esophageal gland genes

**Fig 1:**
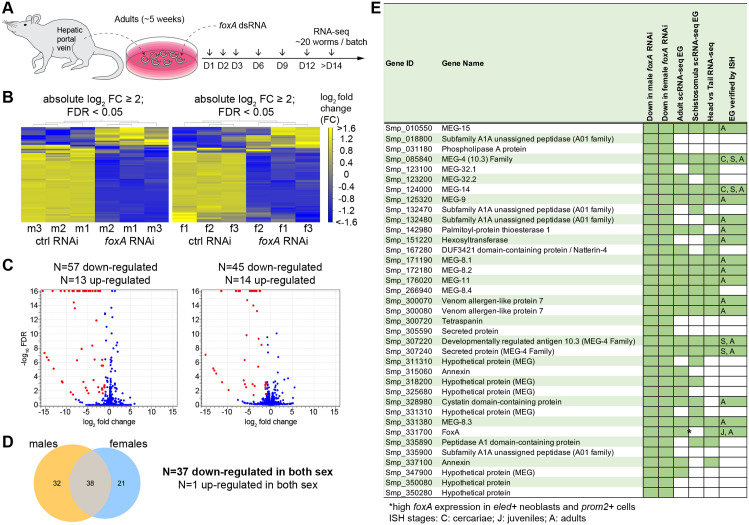
Comparative RNA-seq identifies genes downregulated in *foxA* knockdown parasites. **(A)** Experimental scheme to collect adult *foxA* RNAi parasites for RNA-sequencing. **(B)** Heatmap showing differentially expressed genes in males (left) and females (right). **(C)** Volcano plot of differentially expressed genes in males (left) and females (right). Red dots highlight downregulated genes. **(D)** The Venn diagram shows 38 genes commonly differentially expressed in both sexes (37 downregulated and one upregulated). **(E)** Table of all commonly downregulated genes and their reported EG expression from adult scRNA-seq [11], schistosomula scRNA-seq [19], and head vs tail bulk RNA-seq [14]. Those that are confirmed in their EG expression via *in situ* hybridization (ISH) are indicated with the analyzed stages.

We previously reported that *foxA* (Smp_331700) is enriched in the EG and colocalizes with a known EG gene, *meg-4*, and neighboring *h2b*+ stem/progenitor cells [13]. Adult scRNA-seq revealed that *foxA* is enriched in *eled*+ neoblasts and *prom2* + cells [11], which supports the notion that it acts upstream to regulate stem cell-to-EG cell differentiation. Indeed, *foxA* knockdown results in the apparent loss of the EG tissue, as evidenced by the loss of *meg-4* expression by fluorescence *in situ* hybridization (FISH), the loss of PNA lectin signal, which is highly enriched in the EG, and the downregulation of several known EG genes by qPCR [13]. Therefore, we hypothesized that capitalizing on the *foxA* knockdown worms would reveal genes expressed in the EG tissue. We collected RNA from three batches of adult male (~20 worms per replicate) and female (~30–40 worms per replicate) worms after ~2 weeks of *foxA* knockdown *in vitro* (Fig 1A). The integrity of total RNA was verified, and the knockdown of several known EG genes was confirmed by qPCR (S1 Fig). Differential expression analysis (fold change ≤ -2; false discovery rate ≤ 0.05) revealed significantly downregulated genes in *foxA* knockdown: 57 in males and 45 in females, 37 of which were shared. *foxA* was included in this list, confirming consistent and significant knockdown across all male and female batches (Fig 1E and S1 Table). Based on their annotation, 36 commonly downregulated genes could be divided into six categories: microexon genes (MEGs), enzymes, secreted proteins/toxins, membrane proteins, a protease inhibitor, and hypothetical proteins.

**Fig 2:**
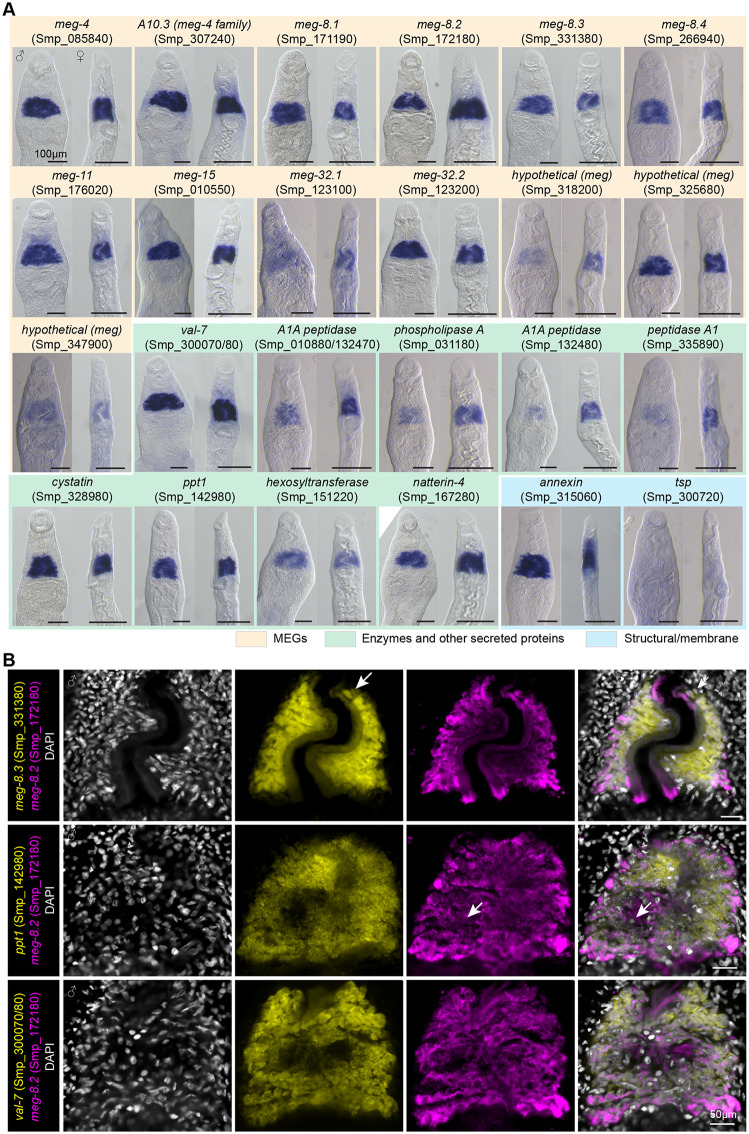
Genes downregulated in *foxA* knockdown are highly enriched in the EG. **(A)** WISH of downregulated genes in adult males and females. Between five and ten worms per sex were analyzed for each gene. **(B)** dFISH of select EG genes reveals subtle heterogeneous expression patterns. A few cells with a noticeable difference in signal between the two channels are indicated by the white arrow. A single confocal z-section from the head region of a male is shown for each combination. Between five and ten males were analyzed per condition.

50% (18 of 36) of the genes were microexon genes (MEGs). The protein-coding portion of the gene comprises microexons that typically range between 6 and 36 base pairs [15,16]. MEGs found in *Schistosomatidae* appear to have no similarity to other microexons in vertebrates [17] or in aphids [18]. Those previously validated by *in situ* hybridization as *bona fide* EG genes (*meg-4* family, *meg-8* family, *meg-9*, *meg-11*, *meg-14*, and *meg-15*) were among the top hits. The other MEGs in the list have also been reported to be expressed in the EG from either bulk [14] or schistosomula/adult scRNA-seq [11,19], but their expression had not yet been validated. 22% (8 of 36) of the genes encode enzymes such as *peptidase*, phospholipase A (*pla*), palmitoyl-protein thioesterase 1 (*ppt1*), and *hexosyltransferase*, most of which have been confirmed to be expressed in EG [14,16,20]. In addition, known secreted proteins/toxins such as venom allergen-like protein 7 (*val-7*) and *natterin-4*, as well as a protease inhibitor *cystatin,* were identified in our dataset. These data suggest that our approach successfully identified the most commonly shared EG genes. We used colorimetric whole-mount *in situ* hybridization (WISH) to validate the EG expression of as many genes as possible (Fig 2A). In addition to known EG genes, we confirmed the enriched EG expression of *meg-32.1*, *meg-32.2*, *meg-8.4*, three other *meg*s (Smp_318200, Smp_325680, and Smp_347900), *natterin-4*, *annexin* (Smp_315060), and two peptidases (Smp_018800 and Smp_335890). *tsp* (Smp_300720) had a barely detectable signal in the EG, which might be due in part to technical challenges in detecting transcripts of low abundance or to its diffuse expression throughout the parasite body, suggesting a potential indirect effect of *foxA* knockdown. Several genes had not been reported in the EG, including *secreted protein* (Smp_305590), *peptidase* (Smp_335900), and two *hypothetical proteins* (Smp_350080 and Smp_350280). However, these genes could not be confirmed by WISH either due to the unsuccessful cloning or the lack of a detectable signal. Outside of 37 shared downregulated genes, 20 were significantly downregulated in males and 8 in females (Fig 1C and 1D). A few of these genes were enriched in the EG, such as *secreted protein* (Smp_331590) and *tsp* (Smp_320440) in both males and females (S2A Fig). Meanwhile, based on the published scRNA-seq atlas [11], other downregulated genes in males and females were enriched in non-EG cell types. For instance, several (4 of 8) genes downregulated in females included eggshell proteins that appear enriched in vitellocytes or eggs (S2B Fig). Similarly, several genes upregulated in *foxA* RNAi males (5 of 13) and females (2 of 14) were also related to reproductive development with vitellocytes and Mehlis gland enrichment. We speculate that these are likely due to the worms’ variable size and reproductive status, and sparse ectopic Mehlis gland-forming males represented in different batches.

Having identified a core set of EG genes, we sought to determine if these genes are directly regulated by FoxA. Due to the lack of a specific antibody against FoxA, we took a bioinformatics approach by analyzing the local enrichment of binding motifs from the known transcription factor (JASPAR) database on the promoter sequences (5 kb upstream) of 36 genes (excluding *foxA*) using the MEME suite [21] (S3A, S3B and S3C Fig). Among the 2430 motifs in the database, only nine motifs, all forkhead transcription factors, showed strong enrichment close to the transcription start site (~130 bp upstream) on 78% (28 of 36) of the promoter sequences. Moreover, the top two enriched motifs were those of human FOXE1 and FOXB1, which are homologous to Smp_342790 (*foxL*) and Smp_331700 (*foxA*). These results suggest a potential role of FoxA in directly regulating the transcription of core EG genes and that the expression of EG genes is likely associated with the differentiation of *foxA*+ stem cells.

To examine the potential heterogeneity of the EG cells, we used double fluorescence *in situ* hybridization (dFISH) of several identified markers (Fig 2B). Specifically, using *meg-8.2* (Smp_172180), we tested co-expression with another MEG (*meg-8.3*, Smp_331380) and two enzymes, *ppt1* (Smp_142980) and *val-7* (Smp_300070/80). The rationales for choosing these markers included the potentially important functions of MEG-8.2 and MEG-8.3 in EG function and maintenance that will become clear in the later part of this manuscript, a non-MEG gene with the highest fold change from RNA-seq (*val-7/8*), and a potential functional connection (*ppt-1*, a depalmitoylating enzyme important for membrane trafficking and lysosomal protein degradation). We noticed a slight variation in signal intensity in some EG cells and a few cells that appeared to have noticeably low levels of one of the transcripts (white arrows), suggesting that heterogeneity may exist among the EG cells. However, virtually all EG cells expressed the analyzed EG markers, indicating that the impact of potential heterogeneity in the EG function might be low. Taken together, by exploiting *foxA* knockdown, we identified several new EG genes and confirmed multiple reported EG genes in their tissue-specific expression. Most identified genes have a conserved forkhead-binding motif in their promoter, suggesting that their expression is likely regulated directly by FoxA.

### FoxA is primarily restricted to the EG cell lineage, and EG loss does not perturb stem/progenitor cell balance

Adult scRNA-seq data suggest that *foxA* expression is highest in *eled*+ neoblasts and *prom2* + intestinal progenitors [11]. Previous work indicates that schistosome stem cells respond to tissue injury or cell death, increasing proliferation to replenish missing cell types [22]. *cbp1*, a CBP/p300 family transcriptional co-activator expressed throughout multiple stem/progenitor cell types, plays a role in this process throughout the body, including the EG. Thus, we sought to better understand the role of FoxA in stem cell-driven homeostasis. We analyzed our dataset to examine expression changes in stem/progenitor cell markers identified thus far in single-cell studies [11,19,23–27] in *foxA* knockdown (S3D Fig). Expression levels of stem/progenitor cells’ subcluster markers, including *ago2–1*, *klf*, *nanos-2*, *fgfrA*, *fgfrB*, *hesl*, *zfp-1*, and *eled,* did not significantly change. Similarly, markers of *eled*-related subclusters (i.e., *astf* and *bhlh*), germline stem cells (*nanos-1*), S1 vitellocytes (*nr/vf1*), and somatic lineage progenitors (tegument: *p53-1* and *zfp-1–1*; gut: *hnf4*; flame cells: *sialidase*) also were not significantly different in *foxA* knockdown worms. *vwa*, a marker of Mehlis gland cell progenitors, was the only marker that was substantially up in *foxA* knockdown males and was significantly down in *foxA* knockdown females. Since *foxA* is not enriched in the Mehlis gland, we speculate that this may be due to variations in the reproductive status of males and females, or to an unknown paracrine role of the EG in reproductive development or maintenance. Levels of *cbp1* were also similar between control and *foxA* knockdown worms. Together, these data suggest that FoxA is primarily restricted to the EG cell lineage, and that EG loss does not significantly perturb the balance or heterogeneity of stem cells during homeostasis, at least during the first two weeks of *in vitro* culture.

### Targeted RNAi screening using *in vitro* leukocyte feeding identifies MEG-8.2 as a key EG factor necessary for lysing ingested leukocytes

**Fig 3:**
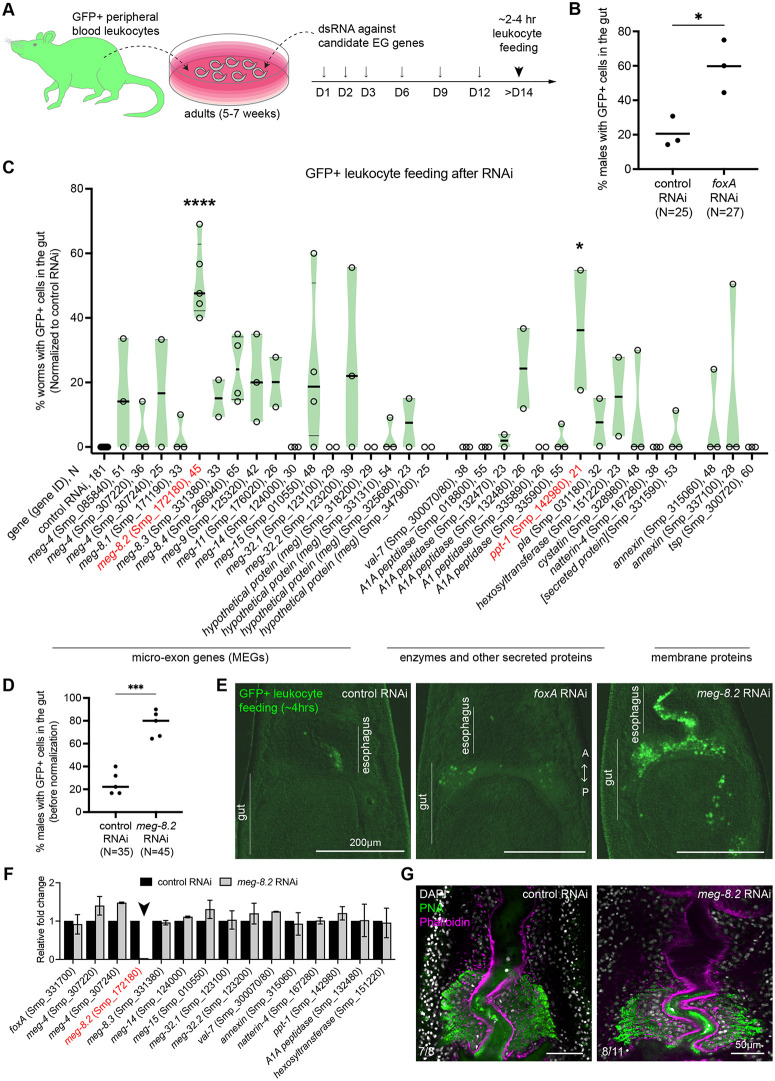
*meg-8.2* knockdown does not affect EG integrity and phenocopies *foxA* knockdown in its failure to degrade ingested leukocytes. **(A)** Experimental scheme to knock down individual EG genes and feed the parasites with leukocytes derived from the peripheral blood of GFP-expressing mice. **(B)** Three independent experiments show an increased percentage of worms (males) with one or more detectable GFP+ leukocytes in the anterior gut lumen. N: total worms from three independent experiments. **(C)** RNAi screen of 32 EG genes. Each dot represents the percentage of worms (males) with one or more detectable GFP+ leukocytes in the anterior gut lumen in each independent experiment. The percentage value was normalized by subtracting the percentage value of control worms in that experiment. Between two and five independent experiments were performed for each gene. N: total worms analyzed in all experiments. An ordinary one-way ANOVA was performed to analyze the statistical significance: *P < 0.05). Smp_305590 (*secreted protein*) failed to clone, and thus, Smp_331590 (*secreted protein*), which is significantly downregulated in males but not in females, was included in the screen instead. **(D)** Unnormalized data for *meg-8.2* knockdown shown in (C). Paired t-test was used to determine the significance: *** P < 0.001. **(E)** Images of the head region show an accumulation of GFP+ leukocytes in the gut lumen of *foxA* and *meg-8.2* knockdown males. **(F)** qPCR of EG genes in *meg-8.2* knockdown showing a specific downregulation of *meg-8.2* and relatively similar levels of other genes. cDNA samples were derived from control and *meg-8.2* knockdown adult males. **(G)** PNA and Phalloidin labeling show that EG appears largely unperturbed. The numbers indicate the male worms analyzed.

Previously, we reported that in the absence of EG, ingested leukocytes fail to be degraded in the esophageal lumen, leading to their accumulation in the gut [13]. This result indicates that one or more EG factors induce rapid lysis of ingested leukocytes within the lumen. Such a function appears intimately linked to parasite survival *in vivo*, since nearly all *foxA* knockdown parasites are cleared from the host vasculature. Therefore, we sought to determine if one or more EG factors would play a dominant role in lysing leukocytes. We knocked down 32 EG genes individually, fed peripheral leukocytes derived from *UBC-GFP* mice [28] to adult worms between 2–4 hours, and quantified the number of males with more than one intact cell in the anterior gut lumen (Fig 3A). We could not reliably quantify females because their range of motion is significantly greater than that of males. Similar to our previous findings, while a basal level of males (~20%) contained GFP+ cells in the gut, *foxA* knockdown males showed a significantly higher fraction of worms (~60%) bearing GFP+ cells in the gut (Fig 3B and 3E). We recorded at least 20 males from two or more independent experiments. To compare differences across gene knockdowns, we normalized each data set by subtracting the control percentage from each data point, setting the control value to zero (Fig 3C). Intriguingly, we found two candidate genes with a significant increase in the fraction of males with GFP+ cells in the gut lumen: *meg-8.2* (Smp_172180) and *ppt-1* (Smp_142980). Of the two, *ppt-1*, although significant, showed greater variability, whereas *meg-8.2* consistently showed an increase across multiple independent experiments (Fig 3D and 3E).

To investigate whether the absence or variability in the feeding phenotype is due to the insufficient knockdown, we assessed the knockdown efficiency of the target gene using qPCR (S4A and S4B Fig). All 32 genes were downregulated by at least 50% relative to the control worms after the knockdown. Specifically, 22 of 32 genes were downregulated by more than 90% relative to the control worms, six by 70–90%, and four by 55–70% (S4C Fig). The four genes with <70% knockdown efficiency included Smp_123100 (*meg-32.1*), Smp_331310 (*meg*), Smp_347900 (*meg*), and Smp_337100 (*annexin*). Among these, the three *meg*s showed only 0–3% of RNAi males with GFP+ cells in the gut after feeding, while *annexin* knockdown males were variable (Figs 3C and S4C). These suggest that insufficient knockdown could be a contributing factor in the phenotypic absence or variability for these four genes. Meanwhile, comparing our cloned sequences of five peptidases (Smp_018800, Smp_132470, Smp_132480, Smp_335890, and Smp_335900), we noticed shared sequence identities (~90% or higher) spanning 200–1000 bp (S4B Fig). Indeed, we observed downregulation of all five peptidases in each gene knockdown, suggesting a cross-targeting effect of the generated double-stranded RNAs on the EG peptidase family genes. Four of five peptidase knockdowns showed 0–3% phenotype (Figs 3C and S4C), suggesting that peptidases are unlikely to degrade ingested leukocytes directly.

To determine whether *meg-8.2* knockdown perturbs EG integrity, we analyzed changes in EG gene expression using qPCR (Fig 3F). All tested EG genes were expressed at levels similar to those of the control worms, suggesting that *meg-8.2* is specifically downregulated. In addition, most *meg-8.2* knockdown worms’ EG was positively labeled by PNA (Fig 3G). These data indicate that *meg-8.2* knockdown does not perturb EG integrity but phenocopies *foxA* knockdown, in which the parasites fail to capture and degrade ingested leukocytes in the esophagus. To assess whether the *meg-8.2* expression level contributes to the degree of leukocyte ingestion phenotype, we analyzed the relative *meg-8.2* expression in genes with high knockdown efficiency (>85%) yet showing high variability (*meg-15*, *meg-32.2*, and *ppt-1*). Although *annexin* (Smp_337100) showed high phenotypic variability, we excluded it from this analysis since its knockdown efficiency was suboptimal (~65% downregulated) (S4A Fig). We found that all three gene knockdowns had reduced *meg-8.2* expression relative to the control (S4D Fig), suggesting that low MEG-8.2 levels may contribute to the penetrance of the phenotype. We speculate that the downregulation of *meg-8.2* in these knockdowns might be due to the defect in EG tissue maintenance. Meanwhile, such a correlation was not observed for MEG-8 family gene knockdowns. Specifically, in *meg-8.1*, *meg-8.3*, and *meg-8.4* knockdowns, while the respective target genes were efficiently knocked down, *meg-8.2* and other EG genes were not significantly downregulated (S4E and S4F Fig). Given that *meg-8.3* and *meg-8.4* knockdowns showed moderate phenotypes (mean: 15% and 24%, respectively) (Fig 3C), these results suggest that MEG-8.3 and MEG-8.4 may also contribute to the blocking or degrading of ingested leukocytes. Together, our RNAi screen revealed that MEG-8.2 may play a dominant role in degrading ingested leukocytes, warranting further investigation into its potential mechanism of action.

### Recombinant MEG-8.2 directly lyses host leukocytes and erythrocytes

**Fig 4:**
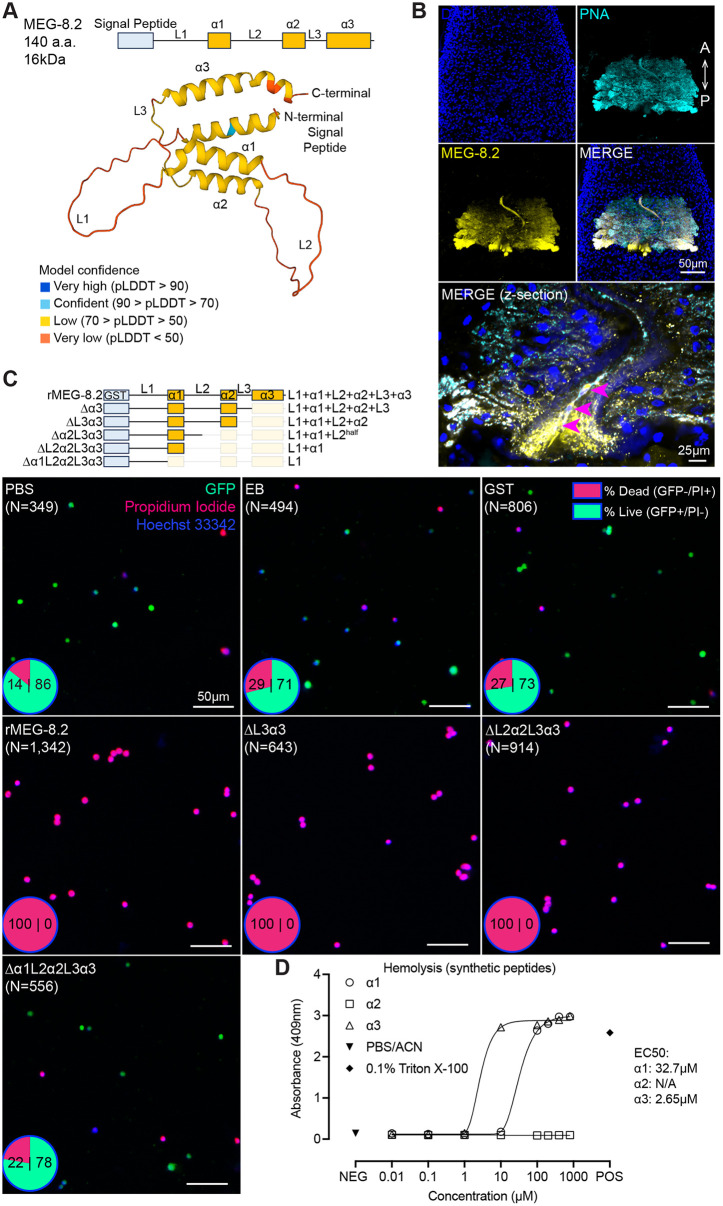
MEG-8.2 helices directly lyse host leukocytes and erythrocytes. **(A)** AlphaFold structure prediction shows an N-terminal signal peptide and three helices spanned by three disordered linkers. **(B)** Confocal image of an adult male head labeled with PNA lectin (cyan) and α-MEG-8.2 (yellow). Maximum Intensity Projection is shown for low-magnification images, and the merged high-magnification image of a different male (below) is shown as a single confocal z-section. Arrowheads show host leukocytes in the esophageal lumen. **(C)** GFP-expressing leukocytes were treated with truncated MEG-8.2 mutants for 10 minutes at 37°C. Each panel shows an overlaid representative image of GFP, PI (magenta), and Hoechst33342 (blue) for controls (PBS, elution buffer, GST only) and mutants. % viability is shown as a pie chart in the lower left corner of each image. The two numbers inside each pie chart are the percentages of dead (PI + /GFP-) and live (PI-/GFP+) cells. N: total number of cells counted. **(D)** Hemolytic activity of synthesized α1, α2, and α3 peptides. α1 and α3 peptides were dissolved in PBS/ACN, and α2 in PBS.

MEG-8.2 is one of the four members of the MEG-8 family [29]. Previous studies identified over 50 transcripts that belong to the MEG family [15,16,29,30]. To better understand the relationship between MEG-8 family genes and other MEGs, we retrieved amino acid sequences of all identifiable MEGs from the *S. mansoni* genome (SM_V10) [15] and analyzed their phylogeny. MEG-8.2 closely aligned with MEG-8.3 (Smp_331380) and MEG-8.4 (Smp_266940), while MEG-8.1 (Smp_171190) was more distant (S5 Fig). As reported, when BLASTed against other members of the *Schistosomatidae* family, it was evident that closely related species such as *S. rodhaini*, *S. haematobium*, *S. japonicum*, and *S. bovis*, as well as more distant avian/animal schistosomes (e.g., *Trichobilharzia*, *Heterobilharzia*), carry several MEG-8-family orthologs (S6A Fig) [29]. MEG-8 family proteins are relatively small, and all have a ~ 20 amino acid N-terminal signal peptide, suggesting that this region is likely cleaved upon secretion. AlphaFold predicts that towards the C-terminus, MEG-8.2 has three helices (α1: 50 – 62; α2: 91 – 103; and α3: 115 – 138 amino acid positions) (Fig 4A). MEG-8.2 amino acid positions E107, P110, W121, L123, F124, F128, and L129 are conserved among the MEG-8 family proteins (S6B Fig). These residues span the disordered linker (L3) between α2 and α3 (104 – 114) and α3 (115 – 138). Previous studies hypothesized that MEGs are alternatively spliced to produce distinct protein variants [15,16]. However, the functional relevance of the potential protein variants remains unclear. *meg-8.2* consists of 17 exons, 15 of which are micro-exons ranging between 12 and 33 base pairs (S6C Fig). To determine the predominant form of splice variants, we generated and sequenced ten independent clones from the total cDNA of adult males, adult females, and day one and day seven schistosomula. Only one out of 10 clones carried a cDNA with a skipped exon 11 (S6C Fig), resulting in a non-frameshift mutation that lacks 10 amino acids (79–88aa) located in the disordered linker (L2) between α1 and α2. However, the rest of the clones were at the full length. While these findings do not rule out the presence of alternatively spliced transcripts, the full-length protein is likely predominantly expressed.

To understand the function of MEG-8.2, we first generated custom α-MEG-8.2 antibodies against a C-terminal epitope, which labeled most of the EG cells (Fig 4B). MEG-8.2 was also detected inside the esophageal lumen and surrounding the leukocytes caught in the lumen. The immunofluorescence signal in the EG could only be detected in control (RNAi) worms, but not in secondary antibody only or *meg-8.2* (RNAi) worms (S7A Fig). We noted that although the antibody readily detects MEG-8.2, it shows some cross-reactivity with MEG-8.3, likely due to the similarity in the C-terminal sequence (S7A and S7B Fig). Together with RNAi screen results (Fig 3), these data suggest that EG-secreted MEG-8.2 directly or indirectly degrades the ingested host leukocytes. To test this hypothesis, we replaced the N-terminal signal peptide with a GST affinity tag and expressed the full-length recombinant MEG-8.2 in *E. coli* (GST-rMEG-8.2, ~ 40kDa) (S7C Fig). Upon induction of *E. coli* harboring an empty plasmid with isopropyl β-D-thiogalactopyranoside (IPTG), GST-rMEG-8.2 (referred to as rMEG-8.2 hereafter) (~40kDa) or GST-only (26kDa) was induced at the correct size band, which was purified using immobilized glutathione beads (S7D and S7E Fig). For rMEG-8.2, we noted several smaller-sized proteins that appear to be degradation products. We also noted that a portion of the induced protein was insoluble. Despite these minor technical challenges, the final eluant after bead purification included full-length rMEG-8.2, confirmed by western blotting using α-MEG-8.2 antibodies.

To determine if MEG-8.2 plays a direct or an indirect role in degrading host leukocytes, we isolated GFP-expressing leukocytes from the peripheral blood of *UBC-GFP* mice, treated the cells with GST-only or rMEG-8.2 for 10 minutes at 37°C, and stained the cells with propidium iodide (PI) and Hoechst (Fig 4C). Cells treated with PBS, elution buffer (EB), or GST retained viability (GFP + /PI-) above 70%. Intriguingly, we observed that virtually all cells treated with rMEG-8.2 had lost cytoplasmic GFP signal and stained positively with PI, indicating that MEG-8.2 can directly lyse the leukocytes. In parallel, rMEG-8.2, but not GST-only or EB control, also displayed hemolytic activity against the whole peripheral blood, indicating that MEG-8.2 is also capable of lysing erythrocytes (S8A Fig). Together, these results support a ‘direct’ role for MEG-8.2 in lysing ingested leukocytes within the esophageal lumen, which is corroborated by its knockdown phenotype (Fig 3).

How does MEG-8.2 lyse host cells? As reported, similar to helices of other MEGs [14,29,31], the three predicted MEG-8.2 helices have a hydrophobicity index between 0.4 and 0.8, with several non-polar residues biased towards one-half of the helical surface, indicating their likely amphipathic nature (S8C Fig). To determine whether any of the three helices contribute to its activity against host blood cells, we generated a series of C-terminal truncation mutations and purified the proteins (Figs 4C, S7G and S8B). rMEG-8.2 mutants lacking α3, L3, α2, and L2 all retained the lytic activity against the isolated leukocytes, suggesting that these domains are dispensable for this activity. The lytic activity was lost only when the deletion included the α1 domain, suggesting that α1 is sufficient and is the minimum domain required to induce cell lysis. Similar results were observed in the hemolysis assay (S8A Fig), indicating that MEG-8.2 α1 might also be directly lysing erythrocytes. To determine if the lytic activity of MEG-8.2 helices is concentration-dependent, we synthesized the three peptides (α1: FWRRMWNSFTSMF; α2: LKERIMNKFNSIF; α3: FTERLWMLFKHCFLNFKNLAKIF) and tested a range of concentrations using hemolysis (Figs 4D and S8D) and leukocyte lysis assays (S9C Fig). In both assays, while α2 did not lyse the cells, α1 and α3 displayed cell lytic activity at micromolar concentrations. Although α3 displayed higher activity than α1, it was dispensable for lysis from our assays using recombinant proteins. Together, these results suggest that while α1 is sufficient for cell lysis, α3 might enhance the cell lytic activity.

To determine whether other MEG-8 proteins exhibit similar activity against host cells, we recombinantly purified MEG-8.1, MEG-8.3, and MEG-8.4 (S7F Fig) and tested them against leukocytes and erythrocytes (S8A and S8B Fig). While rMEG-8.1 and rMEG-8.4 did not lyse the cells, rMEG-8.3 also induced lysis of both leukocytes and erythrocytes. Sequence alignment indicates that MEG-8.2 has more shared residues with MEG-8.3 than with MEG-8.1 or MEG-8.4 (S6B Fig). We note that, as with rMEG-8.2, purified rMEG-8.1 and rMEG-8.4 contained both full-length and smaller proteins (likely representing degradation products), and that we cannot rule out a stronger dependence on the full-length protein for their lytic activity. To determine if the cell lytic activities of the recombinant MEG-8.1/-8.3/-8.4 proteins are due to the specific helical domains, we synthesized peptides corresponding to the MEG-8.2 helices (S9A Fig). MEG-8.3 and MEG-8.4 lacked α1-equivalent domain, but α2- and α3-like domains were present in all three proteins. Consistent with the lytic activity of recombinant proteins, MEG-8.1 and MEG-8.4 peptides did not show any hemolytic activity (S9B Fig). For MEG-8.3 peptides, while α2 had no activity, α3 showed a comparable hemolytic (S9B Fig) and leukolytic (S9C Fig) activities to MEG-8.2 α3. Together, these results suggest that MEG-8.2 α1 and α3, and MEG-8.3 α3 domains are likely responsible for the respective proteins’ host cell-lytic activities.

### The role of MEG-8.2 in parasite tissue homeostasis

Previous findings have shown that knockdown of a closely related MEG-8 family gene, *meg-8.3*, results in local tissue degeneration (i.e., loss of PNA in the EG and increased dextran uptake in the head) [32]. The authors speculated that MEG-8.3 might inhibit other EG factors from targeting the parasite tissues. Given the sequence similarity between MEG-8.2 and MEG-8.3 and their lytic activity against host blood cells, we further investigated the relationship between MEG-8.2 and MEG-8.3, as well as their roles in tissue homeostasis. First, given such a potent activity, we asked whether MEG-8.2 and MEG-8.3 can also lyse parasite cells. To test this, we dissociated adult males into a single-cell suspension, treated them with rMEG-8.2, and used FDA and PI to identify live and dead cells, respectively. While GST-only treated cells contained a mixture of live (FDA + /PI-) and dead (FDA-/PI+) cells, FDA + /PI- cells were absent in rMEG-8.2 treated cells, suggesting that rMEG-8.2 is capable of lysing parasite cells (S10A Fig). In addition, similar to their activity against host blood cells, the three lytic peptides (MEG-8.2 α1/α3 and MEG-8.3 α3) also showed a concentration-dependent reduction in FDA fluorescence, whereas other peptides did not (S9D Fig). Next, to assess their roles in EG tissue maintenance, we quantified the area of the EG relative to the head area in adult males (S10B Fig). In control knockdowns, we noted that the EG area (measured by PNA staining or *meg-8.4* FISH) consisted of ~19% of the head area and showed a good correlation in different-sized males (R^2^ = ~0.7) (S10C Fig). The EG-to-head ratio was maintained in *meg-8.2* or *meg-8.4* knockdown males but showed a significant reduction in *meg-8.3* knockdown worms (S10D, S10E, S10G and S10I Fig), and a relative reduction of worms expressing a high level of *meg-8.4* (S10H Fig). Given the parasite cell lytic activities of MEG-8.2 and MEG-8.3, we speculated that the EG degeneration in the absence of MEG-8.3 is due to MEG-8.2, and that knocking down *meg-8.2* in *meg-8.3* knockdown background might rescue the degeneration phenotype. However, knocking down both *meg-8.2* and *meg-8.3*, either with a total dsRNA amount matching that of the control knockdown (1x dsRNA) or with the same dsRNA amount for each gene (2x dsRNA), resulted in a qualitative increase in worms with no or low *meg-8.4* expression (S10H Fig). Furthermore, double knockdown worms with a detectable *meg-8.4* signal had a reduction in the EG-to-head area ratio similar to that of *meg-8.3* knockdown worms, which were significantly lower than the control or *meg-8.2* knockdowns (S10D Fig). Similarly, *meg-32.2*, another EG marker, also showed a significant reduction in EG-to-head ratio in *meg-8.3* single and *meg-8.2*/*meg-8.3* double knockdowns (S10E Fig), although the signal was still retained (S10I and S10J Fig). Meanwhile, the worm lengths were not significantly different between the knockdowns (S10F Fig). These results are consistent with previous findings that *meg-8.3* knockdown causes local degeneration of head tissues, and that MEG-8.2 may contribute to the inhibitory effects of MEG-8.3 against other EG factors that could otherwise damage the parasite cells and tissues (S10K Fig).

### MEG-8.2 interacts with host cytoplasmic and membrane proteins

**Fig 5:**
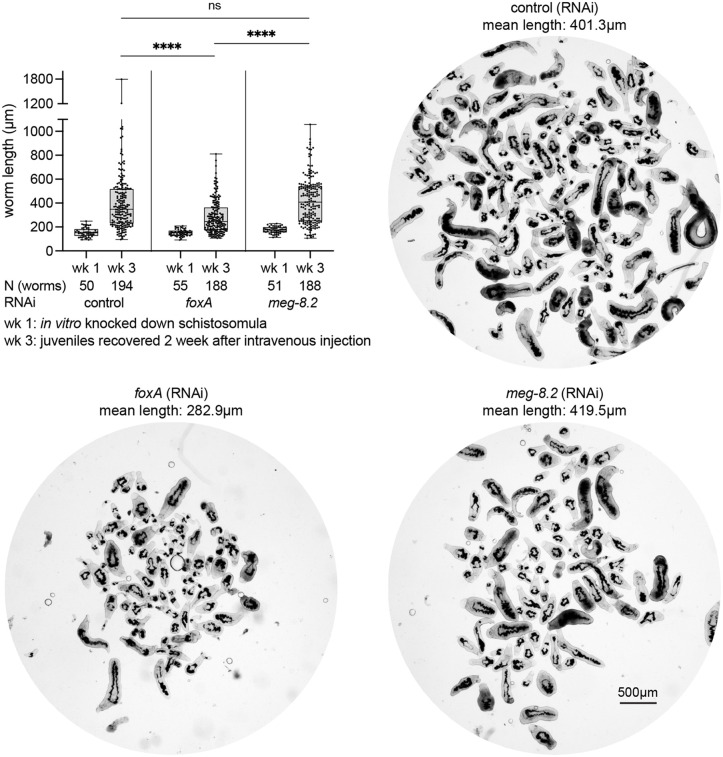
*meg-8.2* knockdown does not perturb *in vivo* parasite development and survival. Juvenile parasites were recovered two weeks after intravenous injection of schistosomula treated *in vitro* with dsRNA for a week. While *foxA* RNAi juveniles are significantly shorter, *meg-8.2* RNAi juveniles have a similar size distribution to control parasites. One-way ANOVA (Tukey’s multiple comparison test) was used for statistical analysis: **** P < 0.0001. N: total number of worms analyzed for length measures. The mean length value for each juvenile group is indicated above each image.

Having identified a key EG factor that blocks and degrades ingested host cells, we sought to tease apart whether such a function is linked to the development and survival of schistosomes *in vivo*. Previously, we showed that when transplanted into a naïve host, adult schistosomes lacking the EG (via *foxA* knockdown) can survive for a week after transplantation [13]. However, their survival drops > 9-fold (57.5% in control males vs. 6.2% in *foxA* knockdown males) 2–4 weeks post-transplantation, and a few remaining survivors are severely stunted. To determine if EG-lacking schistosomes have defects during the development of the parasite and whether the MEG-8.2-driven blood cell lysis impacts the development and survival of juveniles, we mechanically transformed cercariae to schistosomula, divided them evenly into three groups, treated them with control, *foxA* or *meg-8.2* dsRNA for a week, intravenously injected an approximately equal number of schistosomula, and collected the parasites after two weeks via hepatic perfusion (Fig 5). *foxA* knockdown juveniles were significantly shorter either due to the larger worms (that consume more blood) being cleared by the host and/or due to an unknown EG/FoxA-dependent developmental mechanism. We note that this method is limited in its ability to quantitatively assess survival rate due to inherent technical variability (e.g., loading an equal number of parasites and injecting them into the vein). In contrast, *meg-8.2* knockdown juveniles showed a similar size distribution to control parasites, suggesting that other EG factors might play additional roles and that the EG-mediated immune evasion mechanism likely encompasses a broader scope beyond the initial steps of lysing the ingested leukocytes.

**Fig 6:**
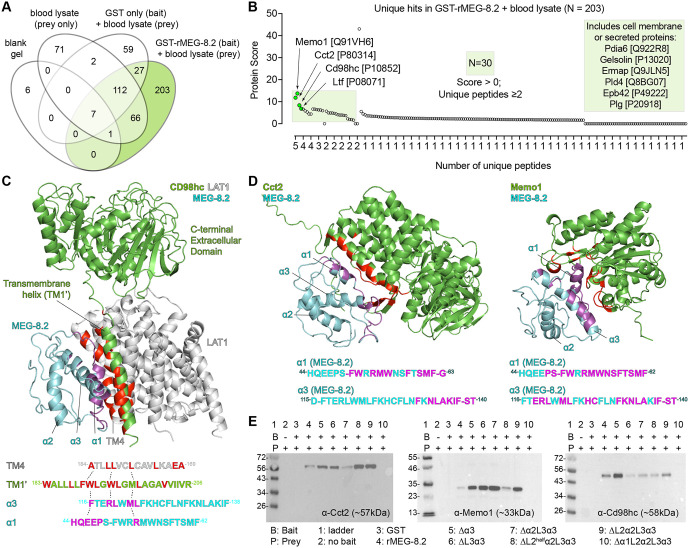
MEG-8.2 interacts with host blood cell proteins. **(A)** The Venn diagram summary shows the number of hits uniquely identified in GST-rMEG-8.2 pull-down (S2 Table). **(B)** Dot plot showing the overall score and the number (N) of unique peptides of the 203 hits. The top four candidates are highlighted with green dots. The light green square includes 30 hits with an overall score above 0 and two or more unique peptides. **(C-D)** Interaction modeling between MEG-8.2 and CD98hc-LAT1 complex [36] is shown in (C), while MEG-8.2-Cct2 and MEG-8.2-Memo1 models are shown in (D). MEG-8.2 residues with an interaction distance <4Å are colored in magenta. Host proteins are labeled in green except for LAT1 (gray), which is not part of the mass spectrometry hits. The counterpart residues of the host protein that interact with MEG-8.2 residues are highlighted in red. **(E)** Western blots of Cct-2, Memo1, and CD98hc in prey (blood lysate) only (lane 1), pull-down samples using GST only (lane 2), or pull-down samples using rMEG-8.2 full lengths and deletion mutants (lanes 3 to 10).

Why do schistosomes secrete large amounts of MEG-8.2 into the lumen to degrade incoming host cells if the ingested cells are likely eventually degraded in the gut lumen, and their degradation by MEG-8.2 appears not essential to the survival of the parasites? We speculated that MEG-8.2 might have additional roles beyond the local lysis of ingested blood cells for two reasons. First, although the individual helices have high hydrophobicity and hydrophobic moment, they are relatively a small part (less than 50%) of the entire sequence – the residues between the helices appear disordered. These regions might assist the helices in interacting with membrane lipids or have a separate role in protein-protein interactions. Such a possibility is substantiated by a previous report of another EG factor (e.g., MEG-14) that was shown to interact with an immunomodulatory protein S100A9 from a yeast two-hybrid screen [33]. Second, known amphipathic helices in other animals play diverse roles, from antimicrobials [34] to receptor binding and signal transduction [35]. Thus, we hypothesized that MEG-8.2 may have a role in parasite-host protein-protein interactions. To identify potential interacting proteins, we used rMEG-8.2 to pull down whole-blood lysate and performed LC-MS/MS (Fig 6A and S2 Table). Excluding false positives by crosschecking any shared hits against the negative controls (blank, prey only, and GST pull-down), we identified 203 hits (UniProt accession IDs), of which 30 had an overall score above zero with two or more unique peptides (Fig 6A and 6B). The top candidates (sorted based on the overall score) among them included Cct2 [P80314], Memo1 [Q91VH6], Cd98hc [P10852], and Ltf [P08071]. To confirm the specificity of these interactions, we performed Western blots of candidate proteins in GST-only and rMEG-8.2 pull-down samples (Fig 6E). We observed bands corresponding to the expected sizes of the host proteins in the rMEG-8.2 pull-down but not in the GST-only pull-down, suggesting that these are true positive interactions.

**Fig 7:**
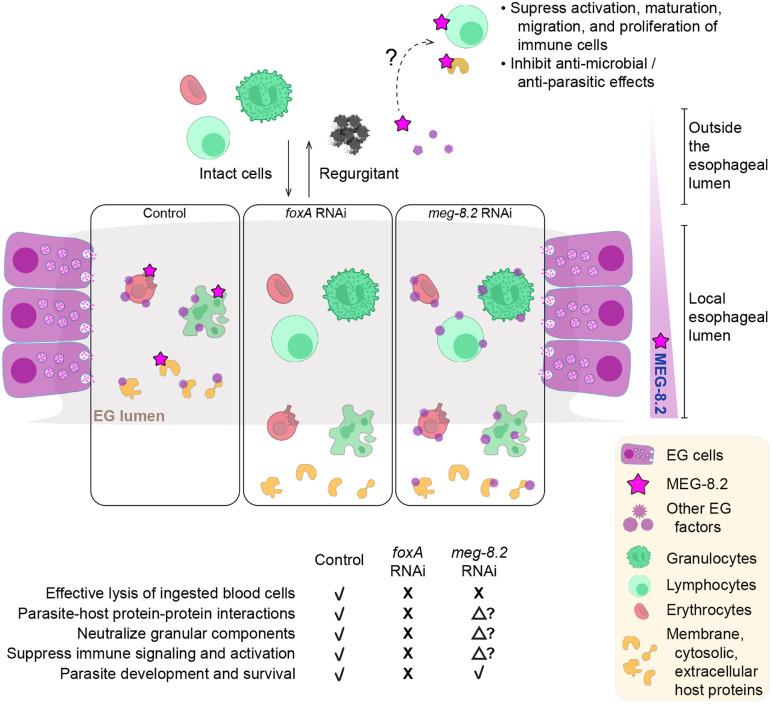
Working model for MEG-8.2-mediated host immune evasion. Model for MEG-8.2-mediated host immune evasion. In control parasites, released EG factors effectively lyse the ingested host cells in the lumen and interact with host proteins that promote parasite survival. MEG-8.2 may also be released into the bloodstream via worms’ regurgitation to interact with host extracellular and membrane proteins to suppress the activation of immune cells. In the absence of EG, no EG factors exist to lyse the cells or interact with host proteins, leading to perturbed parasite development, homeostasis, and survival. In MEG-8.2 absence, host cells are inefficiently degraded, and interactions between other EG factors and host proteins are largely unaffected. The model was generated using Illustrator (Adobe). The intact red blood cell, lysed red blood cell, lymphocyte, and granulocyte images were derived from NIH BioArt (Image number 444, 445, 173, and 141, respectively).

To determine if any of the helices or the disordered regions are required for the interactions, we first took an *in silico* approach by modeling the MEG-8.2 interaction with the candidate proteins (Figs 6C, 6D and S11A). For example, using published structural data [36], we modeled MEG-8.2 with CD98hc (also known as Slc3a2), a transmembrane protein that is reported to form a heterodimer with Slc7 family transporters and functions as an amino acid transporter in integrin signaling and adaptive immunity [37–40]. Several hydrophobic residues of α1 and α3 showed close interaction with the hydrophobic residues of the CD98hc transmembrane helix (TM1’) (Fig 6C). Hydrophobic residues of TM1’ (F189, W193, M196, L197, A200, I203) that normally interact with hydrophobic residues of LAT1 (also known as SLC7A5) TM4 (L173, L177, L181) are closely positioned with the hydrophobic residues of MEG-8.2 (e.g., α3 F116, L120, M122, L123), suggesting that MEG-8.2α1/α3 -TM1’ interaction might interrupt the CD98hc-LAT1 interaction. Similarly, hydrophobic residues of α1 and α3 showed close interactions with Cct2 and Memo1 (Fig 6D) as well as Ltf (Lactotransferrin) (S11A Fig), a known component of neutrophil secretory granule, with antimicrobial properties [41–43]. The western blot of host proteins after a pull-down with the truncation mutants revealed that α3 was dispensable for these interactions, while α1 was necessary (Fig 6E). Furthermore, protein lysate from the plasma of the infected host contained MEG-8.2 but not that of the uninfected host (S11B Fig), suggesting that MEG-8.2 is likely released into the blood. These results suggest that MEG-8.2 α1 plays a dual role: concentration-dependent lysis of the host blood cell membrane and interaction with host proteins involved in the proper function of immune defense. This proposes an interesting possibility that at high (i.e., cell lysing) concentrations within the EG lumen, MEG-8.2 contributes to the effective degradation of incoming blood cells. In contrast, MEG-8.2 released into the host bloodstream (presumably via worms’ regurgitant) is at low concentrations (that cannot lyse cells) but interacts with host cell membrane or secreted/excretory proteins, thereby inhibiting the signaling and activation of the immune system (Fig 7).

## Discussion

As schistosomes ingest large amounts of blood during growth and homeostasis, diverse host cells and proteins enter the digestive tract. These host components can presumably be digested when neutralized and used as a potential nutritional source for the parasites. If not, many components could damage the parasite tissue, either directly by releasing proteases and peroxidases (e.g., granulocyte secretion) that could be damaging, or indirectly by relaying signals to activate, mobilize, and recruit more host immune factors and/or cells. Thus, as the frontline defense, the EG must not only degrade incoming host cells but also neutralize any proteins that could harm the parasites. EG factors have been gaining attention over the past two decades; however, the complete picture of the EG-expressed genes and their functions has remained elusive. Having discovered the essentiality of FoxA in EG cell production [13], using *foxA* RNAi RNA-seq, we were able to identify known and new EG factors. The identified factors are expressed in most, if not all, EG cells without substantial heterogeneity, and many are shared between male and female schistosomes.

From a targeted RNAi screen, we discovered that depletion of MEG-8.2 leads to an accumulation of leukocytes in the gut. While previous studies have hypothesized that MEGs could confuse the host immune system by producing protein variants through exon skipping [12,16], we find that most MEG-8.2 may exist in full-length form. We note that, because our functional screen analyzes a short window of time after initiating feeding, it may not identify all candidates that play a role in lysing ingested leukocytes. Indeed, while MEG-8.2 displayed the most severe phenotype in our assay, other EG factors showed moderate phenotypes, including several MEGs (e.g., MEG-4, MEG-8.3, MEG-8.4, MEG-9, MEG-11, MEG-15, and MEG-32.2) and enzymes (e.g., A1A Peptidase, Ppt-1, and Hexosyltransferase). We also note that the screening pipeline does not pick up parasites with altered physiological or cellular changes associated with each gene knockdown. Recent work suggests that *meg-8.3* knockdown results in EG degeneration over time, rendering the parasites sick [32]. Indeed, we also found that knocking down *meg-8.3* perturbed EG tissue homeostasis, which could not be rescued by knocking down *meg-8.2* (S10B–S10K Fig). Although, in our hands, most of the *meg-8.3* knockdown worms remained attached to the culture dish at the time of feeding, it is possible that they did not ingest as many leukocytes as *meg-8.2* knockdowns. However, a single gene knockdown of *meg-8.2* that results in leukocyte gut accumulation in a significant proportion of worms suggests that MEG-8.2 might be the dominant driver of this process.

Our biochemical investigations using recombinant MEG-8.2, as well as synthetic peptides, reveal the crucial role of individual helices in lysing host leukocytes and erythrocytes. While α1 is the minimal region needed for this activity, α3 is ~ 10 times more potent, and thus, the two domains likely work together to efficiently lyse host cells. Both helices have amphipathic properties (S8C Fig). Amphipathic helices are found in diverse biological processes and play significant roles in membrane curvature sensing, remodeling, and permeabilization [44–46]. Thus, the hydrophobic side of MEG-8.2 α-helices likely insert between the acyl side chains of the lipid bilayer of the host cell membrane, altering membrane curvature and resulting in permeabilization. In addition, as reported for other MEGs (e.g., MEG-14) [47], MEG-8.2 linkers that connect the helices appear disordered (Fig 4A). These internally disordered regions fold differently under varying environmental conditions, such as elevated temperature, the presence of negatively charged lipids/detergents, and dehydration, suggesting that they may contribute to the context-dependent roles of MEG-8.2 in targeting different cells and molecules. Meanwhile, previous studies report that secondary structure prediction of several MEGs (e.g., MEG-9, -12, -19, -24, -26, -27, and -28) reveal that they also have amphipathic helices [14,29,31]. This is consistent with the fact that MEG-8.3 has a C-terminus with a high sequence similarity to MEG-8.2 α3 (S6B Fig), which is likely responsible for its lytic activity (S8A, S8B, S9B and S9C Figs). Given that several MEGs show a moderate increase in leukocyte gut accumulation from our functional screen (Fig 3C), it will be important to tease apart the relative contributions of amphipathic helices of other MEGs in degrading ingested blood cells.

The fact that depletion of MEG-8.2 alone does not appear to have a major impact on parasite development or survival, but that it interacts with multiple host proteins, has important implications. Cell lysis by the EG factors is likely one of the first steps in neutralizing host components. For instance, permeabilized granulocytes may release several factors, such as proteases and peroxidases packed in cytotoxic granules. Neutralizing or sequestering these components in a timely fashion would minimize damage to the parasites. We speculate that although MEG-8.2 is the dominant driver of host cell lysis in the esophageal lumen, different EG factors interact with distinct host proteins to inhibit their activity before they are sent into the gut. This model is supported by the fact that MEG-14, another prominent EG factor, interacts with S100A9 [33], a calgranulin protein expressed in granulocytes (e.g., neutrophils) that promotes and regulates inflammatory processes [48,49]. According to this model (Fig 7), in *foxA* knockdown, all EG factors are absent, and therefore, the parasites likely accumulate damage over time and can no longer survive. However, other EG factors are still present in *meg-8.2* knockdown (such as MEG-8.3, which, in its recombinant form, can also lyse host cells), which is why parasites are not affected. This model also explains why other attempts to inhibit a single EG factor (e.g., MEG-4) have not resulted in a dramatic increase in parasite clearance [50].

Our comparative mass spectrometry of rMEG-8.2 pull-down reveals that it interacts with various host membrane, cytosolic, and extracellular proteins. Cct2 (T-complex protein 1β), a component of the chaperonin-containing T-complex (TRiC), is a cytoplasmic protein involved in protein folding and proteostasis [51,52] by acting as an aggrephagy receptor, binding to misfolded aggregation-prone proteins for clearance [53]. Memo1 (mediator of ErbB2-driven cell motility 1), another cytoplasmic protein, promotes cell proliferation and migration [54] and binds to Copper (Cu), serving as a redox enzyme to sustain O_2_^–^ production by NADPH [55,56]. Cct2 and Memo1 are found in extracellular vesicles [57–61], suggesting that their interaction with MEG-8.2 may occur outside of the host cell. Whether these interactions occur inside (e.g., MEG-8.2 being phagocytosed or entering the permeabilized cell’s cytoplasm) or outside (e.g., released from permeabilized cells or via extracellular vesicles) the host cell remains to be determined. In either case, MEG-8.2’s interaction with these proteins may have functional relevance that is advantageous to the parasites (e.g., preventing protein aggregation or lowering oxidative stress). CD98hc (amino acid transporter heavy chain SLC3A2) is a membrane protein with a single transmembrane (TM1’) helix and a C-terminal extracellular domain. As modeled (Fig 6C), MEG-8.2 α1 and α3 preferentially interact with TM1’ via hydrophobic interactions with residues that are important in forming a heterodimer with SLC7 protein [36]. CD98hc facilitates the integrin-dependent proliferation of T and B lymphocytes [40,62]. MEG-8.2 presence in blood plasma suggests that it is excreted from parasites. The excreted MEG-8.2 (at low concentration) may interact with CD98hc-expressing T and B cells (e.g., bloodstream or peripheral lymphoid organs) to inhibit their activation/proliferation (Fig 7). Lactotransferrin (Ltf) binds to iron and is found in serum and secretory fluids (e.g., breast milk, saliva) and secretory granules of neutrophils [41,43,63]. It also has antiviral, antimicrobial, antifungal, and antiparasitic activity [64,65]. Furthermore, Ltf binds to various receptors (e.g., lipoprotein-related receptor) and is internalized, thereby activating innate and adaptive immune cells [42]. Similarly, further down the list of MEG-8.2 interacting proteins is Gelsolin (S2 Table), which is another neutrophil granular component [43]. These results suggest that MEG-8.2 potentially interacts with granular proteins via hydrophobic interactions of α1 (and α3) to prevent them from damaging the parasites and inhibit their signaling and activation of innate and adaptive immunity.

We note the limitation of our pull-down mass spectrometry, which uses lysed cells to probe protein-protein interactions *in vitro*. Thus, future investigations are necessary to validate the discovered interactions *in vivo*, and to dissect the potential role and the mechanism of MEG-8.2-host protein interactions in suppressing host immunity. In addition, devising novel *in vitro* approaches to identify the roles of EG factors beyond lysing ingested leukocytes will be crucial for broadening our understanding of their functional relevance to host immune evasion. A recent study using epitope mapping of proteins expressed in the digestive tract and tegument reveals potential in designing multi-epitope vaccine constructs that can serve as monoclonal antibody targets [66,67]. Several EG MEGs are considered among the top candidates, suggesting their therapeutic potential. In this regard, uncovering the host interaction partners of other EG factors and identifying a combination that causes a significant *in vivo* parasite clearance will have important implications in devising new approaches to target schistosomes.

## Materials and methods

### Ethics statement

Vertebrate animals were handled in accordance with the approved protocols from the Institutional Animal Care and Use Committee (IACUC) at the University of Wisconsin–Madison (M005569) and the Animal Welfare Committee (AWC) at the University of Texas Health Science Center at Houston (AWC-21–0069 and AWC-24–0073).

### Animal care and handling

Swiss-Webster mice (Hsd: ND4, Envigo or SW-F, Taconic Biosciences) between 3 and 5 weeks of age were infected with *S. mansoni* (NMRI strain) received from the Biomedical Research Institute (Rockville, MD). Infected mice were harvested via hepatic perfusion [68] between 5 and 7 weeks to collect adult parasites. Peripheral blood was collected from Swiss-Webster, C57BL/6J (#000664, The Jackson Laboratory), or *UBC-GFP* (#004353, C57BL/6-Tg(UBC-GFP)30Scha/J, The Jackson Laboratory). For infection with schistosomula, female Swiss-Webster mice, age three to five weeks were used. Mice were weighed and randomly assigned to three treatment groups until infection. Prior to infection, mechanically transformed schistosomula were treated *in vitro* with 15ng/µL of control, *foxA*, and *meg-8.2* dsRNA five times for a week. RNAi schistosomula were washed three times in 1x PBS (Calcium and Magnesium free), and ~3,000 larvae were loaded into a 27G insulin syringe (BD) in ~200µL maximum volume. Following anesthesia using isoflurane (6679401725, Piramal Critical Care), mice were placed laterally, and schistosomula were injected retro-orbitally after confirming aspiration of needle placement in the retro-orbital sinus. Post-inoculation, mice were housed in groups and monitored daily. Mice were euthanized three weeks post-infection using Fatal-Plus (Vortech)/ Heparin (McKesson) cocktail, and worms were recovered via hepatic perfusion.

### RNA interference

Previously published methods were used to synthesize double-stranded RNA for all genes reported in this study [13,69]. S3 Table lists all oligonucleotides used for dsRNA synthesis. Briefly, ~ 15–20 adult parasites were cultured in ABC media [70] without the red blood cells for ~2 weeks *in vitro*. Parasites were treated with dsRNA at 10 – 20µg/mL 6 times during the culture period. For double knockdown of *meg-8.2* and *meg-8.3*, control dsRNA was used at 15µg/mL while each *meg-8.2* and *meg-8.3* dsRNA was used at 7.5µg/mL (1x) or 15µg/mL (2x). Knockdown adult worms were used for RNA-seq, *in situ* hybridization, qPCR, staining, and leukocyte feeding. For schistosomula knockdown, mechanically transformed schistosomula [68,71] were split equally into three conditions (control, *foxA*, *meg-8.2*) and cultured for a week while being treated with dsRNA 4–5 times. Knockdown schistosomula were injected retro-orbitally, and juveniles were collected 2–3 weeks after the injection.

### Quantitative real-time PCR

Total RNAs from knockdown parasites were extracted using TRIzol (15596026, Invitrogen)/phenol-chloroform. Extracted RNA samples were converted to cDNA using the iScript cDNA synthesis kit (Bio-Rad). qPCR was performed using the CFX Real-Time PCR System (Bio-Rad). Relative fold changes were analyzed using the ΔΔCt method. Primers used for qPCR are listed in S3 Table.

### RNA-seq analysis

RNA sequencing (2 x 150 bp, ~ 30 million reads per sample) was performed at the University of Wisconsin Biotechnology Center (Madison, WI). Sequenced reads were processed and analyzed using CLC Genomics Workbench (Qiagen) under default settings. Quality passed reads were mapped to the *S. mansoni* genome (SM_V9 assembly). Differential expression analysis was performed to determine statistical significance (false discovery rate ≤ 0.05; absolute Log_2_ fold change ≥ 1). Raw and processed reads have been deposited in NCBI (GSE278682).

### Parasite labeling and imaging

Colorimetric and fluorescent *in situ* hybridizations were carried out using previously described methods [13,19,26,69,72,73]. Briefly, adult parasites were treated with 2.5% tricane (ethyl 3-aminobenzoate methanesulfonate) to separate the males and females and killed with 0.6M MgCl_2_ before fixing in 4% formaldehyde/PBSTx for ~4 hrs. Fixed worms were dehydrated in methanol and stored at -20ºC. Rehydrated worms were bleached in 0.5% formamide/1.2% H_2_O_2_/0.5x SSC for 45 – 60 minutes under bright light and treated with 10µg/mL Proteinase K (Invitrogen)/0.1% SDS for 30 minutes. Riboprobes were synthesized by *in vitro* transcription. Candidate gene fragments inserted into pJC53.2 were amplified by PCR and were used as a template for a transcription reaction containing either Digoxigenin-11-UTP (DIGUTP-RO, Roche) or Fluorescein-12-UTP (11427857910, Roche) and either T3 or SP6 polymerase, depending on the orientation of the insert, to generate antisense riboprobes. Purified riboprobes were hybridized to parasites at 52 ºC overnight. Worms were incubated with anti–DIG-AP (11093274910, MilliporeSigma) for colorimetric, and anti-DIG-POD (11207733910, MilliporeSigma) or anti–FITC–POD (11426346910, MilliporeSigma) for fluorescent in situ hybridization at 1:1,000 – 1:2,000 dilution overnight. Peanut Agglutinin tagged with fluorescein (VectorLabs) was used at 1:500 to label the esophageal gland. Polyclonal anti-MEG-8.2 antibodies were custom-ordered commercially (ThermoFisher Scientific) by injecting a short C-terminal peptide (EEYNPPKDSDFTER) into a rabbit host. The custom antibody purification followed the vendor’s standard 90-day protocol, which included a primary immunization (250µg) at day 1, first, second, and third immunization boosts (100µg) at days 14, 42, and 56, respectively, an ELISA assay of the collected serum samples, and affinity-purified ELISA titers. Affinity-purified anti-MEG-8.2 antibodies were used at 1:500 for immunofluorescence staining in FISH blocking solution. Secondary goat anti-rabbit IgG (H + L) tagged with Alexa Fluor 568 (A-11036, ThermoFisher Scientific) was used at 1:500 in FISH blocking solution. Low-resolution colorimetric and fluorescent images were taken using Leica M205FCA stereoscope or AxioZoom.V16 stereomicroscope (Carl Zeiss). For high-resolution images, Andor WDb spinning disk confocal microscope (Andor Technology) or Nikon A1 confocal microscope. ImageJ or ImarisViewer (Bitplane) was used to make linear adjustments to brightness and contrast. Schistosomula and juvenile RNAi worms were imaged using ECHO Revolve (BICO).

### *In vitro* leukocyte feeding

~15 – 20 adult parasites were knocked down for individual EG genes for two weeks *in vitro*. Isolated total leukocytes from one to two *UBC-GFP* mice were resuspended in 0.5 – 1 mL of cold 1x PBS. Between 50 – 150µL of leukocyte suspension was added to each knockdown. The volume of cells added to each well differed slightly across each experiment (due to the differences in the total number of gene knockdowns in a particular experiment). However, within each experiment, an equal volume of cells was added to each well. After two to four hours of incubation at 37°C/5%CO_2_, parasites were treated with 2.5% Tricane (ethyl 3-aminobenzoate methanesulfonate, E10521, MilliporeSigma) to separate the males from females and to paralyze them for imaging. Male parasites were placed on a glass slide, and a cover glass was placed to flatten the worms and to prevent drying. The anterior portion of the male worms was imaged using a stereoscope (Leica Microsystems). Parasites with at least one detectable GFP+ cell in the gut lumen were counted towards a positive phenotype (i.e., defective lysis in the esophagus).

### Motif analysis

To investigate if FoxA binds to upstream regions of the identified EG genes, we used the CentriMo (v5.5.5) tool of the MEME Suite (https://meme-suite.org/meme/index.html) [21]. We extracted and compiled the sequences of 5,000 bp upstream of the transcription start site of all 36 EG genes and used them as the input for the CentriMo analysis to identify motifs enriched in these sequences. Command lines: centrimo --oc. --verbosity 1 --local --score 5.0 --ethresh 10.0 --bfile sequences.fa.bg sequences.fa motif_db/JASPAR/JASPAR2022_CORE_redundant_v2.meme.

### Cloning, expression, and purification of recombinant proteins

Full-length rMEG-8.2 and its deletion mutants, as well as other MEG-8 proteins (i.e., MEG-8.1, MEG-8.3, MEG-8.4), were amplified by PCR from *S. mansoni* cDNA. Primers included *BamHI* and *XhoI* restriction sites at 5’ and 3’ ends, respectively. The PCR products and pGEX4T-3 expression vector were digested with *BamHI* and *XhoI*, and the PCR products were purified using a gel DNA recovery kit (D4007, Zymo Research). The digested vector and PCR products were ligated using T4 DNA ligase (M0202L, NEB). The ligation mixtures were transformed into *E. coli* DH5α competent cells. Positive clones were selected on LB agar plates containing ampicillin (100 µg/mL). Plasmid DNA was isolated using a plasmid mini-prep kit (D4020, Zymo Research) and confirmed by restriction digestion and plasmid sequencing (Plasmidsaurus). Verified constructs were transformed into *E. coli* BL21(DE3) cells for protein expression. The plasmid sequences for all constructs used for recombinant protein expression are listed in S4 Table. A single colony from the transformation was used to inoculate 10 mL of LB broth containing ampicillin (100 µg/mL), which was grown overnight at 37°C with shaking. This culture was used to inoculate 500 mL of LB broth with ampicillin (100 µg/mL), and the culture was grown at 37°C until the OD600 reached 0.6-0.8. Protein expression was induced with 0.1 mM IPTG, and the culture was incubated at room temperature for 5 hours. Cells were harvested by centrifugation at 5000 x g for 10 minutes at 4°C. The cell pellet was resuspended in GST-fusion protein lysis buffer (0.1% Triton X-100 in PBS with 1x protease inhibitor). The suspension was sonicated on ice using a large-tip probe (1 min 40 sec total sonication time; 5 sec ON, 10 sec OFF; amplitude at 30%). The lysate was cleared by centrifugation at 20,000 x g for 10 minutes at 4°C, and the supernatant containing the soluble GST-tagged protein was collected. The supernatant was incubated with Glutathione Sepharose 4B beads (17075601, Cytiva) at 4°C for 30 minutes with gentle rotation. The beads were washed three times with GST high wash buffer (0.5 M NaCl, 0.1% Triton X-100 in PBS) and two times with GST lysis buffer. The GST-tagged protein was eluted using elution buffer (50 mM Tris-HCl, pH 8.0, 10 mM reduced glutathione). Protein concentration was determined using the BCA assay kit (23227, Thermo Scientific). Fractions containing the target protein were concentrated and buffer-exchanged into PBS to remove residual glutathione using Amicon columns (UFC501096, Millipore Sigma).

### SDS Gel electrophoresis and Western blotting

Cleaned and concentrated protein fractions were boiled for 10 minutes in 5x SDS-PAGE sampling buffer. The eluted proteins were separated by SDS-PAGE and stained with Coomassie dye for visualization. For western blot analysis, 20 µg of protein samples were loaded onto a gel and transferred onto a PVDF membrane after electrophoresis. The membranes were blocked for 2 hours at room temperature in 1% blocking solution (1% Western Blocking Reagent (WESTBL-RO, Roche) in TBS) and followed by an overnight incubation at 4°C with anti-MEG 8.2 polyclonal antibody (1:2000 dilution). The next day, the membranes were washed three times for 10 minutes each with TBS-Tween wash buffer, then incubated with HRP-conjugated anti-rabbit IgG (32460, Invitrogen) for 1 hour at room temperature. After three additional 15-minute washes, the membranes were developed using the ECL-2 Western Blotting Substrate (80196, Thermo Scientific) and visualized by ChemiDoc MP Imaging System (Bio-Rad).

### Leukocyte lysis

Peripheral blood collected from *UBC-GFP* mice was centrifuged at 500 x g for 5 minutes at 4°C. For each independent experiment, one to two *UBC-GFP* mice were used. The supernatant was removed, and 3 mL of ACK lysis buffer (A1049L-01, Gibco) was added for 3 minutes (room temperature) to lyse the red blood cells. After lysis, the cells were pelleted by centrifugation at 500 x g for 5 minutes at 4°C. The pellet was washed with cold 1X PBS, and the lysis and wash steps were repeated. After the final wash, the cells were resuspended in 500 µL of 1X PBS. Recombinant MEG-8 proteins (full-lengths and mutants) were incubated with leukocytes at a concentration of 0.5 µg/µL in a total reaction volume of 20 µL in a 96-well microplate. Negative controls, including pGEX4T-3 vector-only protein (GST only) and elution buffer only, were used. The cells were incubated for 10 minutes at 37°C. Following incubation, the cells were stained with Hoechst (50 µg/mL) and propidium iodide (1 mg/mL) and imaged using M205FCA stereoscope (Leica microsystems). All MEG-8 peptides were custom-ordered from Biomatik at 95% purity. The peptide sequences are listed in S3 Table and S9A Fig. Lyophilized peptides were dissolved in DMSO, 30% acetonitrile (ACN), 15% ACN, or PBS, depending on solubility. Peptides were added at final concentrations ranging from 1 nM to 800 µM and incubated with leukocytes, bringing the total reaction volume to 150 µL in a 96-well clear-bottom, black-walled plate (Costar). Cells were stained with propidium iodide (1 mg/mL). The plate was incubated at 37 °C for 10 minutes, after which fluorescence was measured at 485nm (excitation)/ 535nm (emission) and 544nm (excitation)/ 612nm (emission) using a plate reader (Synergy H1, 20090225). 30% ACN/1 × PBS or 1 × PBS served as negative controls, and 0.1% Triton X-100 was used as a positive control.

### Parasite cell dissociation and lysis

The dissociation protocol was adapted from Wendt et al., 2020 [11]. In brief, adult male parasites were cut in half and separated into head and tail fragments on ice and rinsed twice with cold 1X PBS. 1 mL of Trypsin-EDTA + 3 mL of PBS were added to each tube. Samples were pipetted constantly with a P1000 pipette for approximately 10 minutes at room temperature. 8 mL of cold Basch media was added to stop the digest, and samples were then spun at 500 rcf for 10 minutes at 4°C. Pellets were resuspended in 1 mL of cold Basch media and filtered through a 100 µm cell strainer. 10 µL/mL RQ1 DNase was added to each sample and incubated for 10 minutes at room temperature. 3 mL of cold 1X PBS + 0.5% BSA was added to each sample, and samples were then centrifuged at 500 rcf for 10 minutes at 4°C. The samples were then resuspended in cold 1X PBS + 0.5% BSA. GST-rMEG-8.2 (full-length) was incubated with cells at a concentration of 0.1 – 0.5 µg/µL in a total reaction volume of 20 µL in a 96-well microplate. Negative controls, including protein from the pGEX-4T-3 vector (GST only), were also used. The cells were incubated for 10 minutes at 37°C. Following incubation, the cells were stained with Fluorescein Diacetate (0.5 mg/mL), Hoechst (50 µg/mL), and propidium iodide (1 mg/mL), and imaged using M205FCA stereoscope (Leica Microsystems). MEG-8.1, MEG-8.2, MEG-8.3, and MEG-8.4 peptides were incubated with cells at concentrations ranging from 10 nM to 800 µM in a total reaction volume of 75 µL in a 96-well clear-bottom, black-walled plate (Costar). Cells were stained with fluorescein diacetate (0.5 mg/mL), Hoechst (50 µg/mL), and propidium iodide (1 mg/mL). The plate was incubated at 37 °C for 10 minutes, and fluorescence was measured at 485nm (excitation)/ 535nm (emission) and 544nm (excitation)/ 612nm (emission) using a plate reader (Synergy H1, 20090225). 30% ACN/1 × PBS or 1 × PBS served as negative controls, and 0.1% Triton X-100 was used as a positive control.

### Hemolysis

Blood collected from C57BL/6 mice was centrifuged for 5 minutes (500g, 4°C). After the centrifugation, the supernatant was removed, and the packed blood cells were washed twice with 10 mL of cold 1x PBS. The cells were then diluted to 1% in 1x PBS, and 75 µL was aliquoted into a 96-well microplate. Controls included pGEX4T-3 vector-only protein (GST, negative control), PBS (negative control), and 0.1% Triton X-100 solution (positive control). MEG-8 full-length and mutant proteins were diluted to 0.5µg/µL in PBS, bringing the total reaction volume to 150 µL. The plate was incubated at 37°C for 10 minutes, then centrifuged at 500 x g for 5 minutes at 4°C. The supernatant was separated and transferred to a new well. Hemoglobin was detected in the supernatant by measuring absorbance at 409 nm using a plate reader (BioTek). Synthetic MEG-8 peptides were also tested with peripheral red blood cells. Peptides were added at concentrations ranging from 0.01 µM to 800 µM. 30% ACN/1X PBS or 1X PBS was used as a negative control, and 0.1% Triton X-100 as a positive control.

### Pull-down

A Pierce GST Protein Interaction Pull-Down Kit (21516, ThermoScientific) was used for the pull-down assay. 100–150µg of the full-length MEG-8.2 and the mutants were used as bait proteins. Blood lysates from the peripheral blood of infected mice were used as prey proteins. For negative controls, pGEX4T-3 vector-only protein (i.e., GST-only) was used as the bait protein, while blood lysates without a bait served as the negative control. The pull-down samples were processed following the kit protocol, and the protein eluates were quantified using a BCA Assay. Subsequently, 40 µg of each sample was loaded onto SDS-PAGE gels, which were stained using silver staining. The staining procedure involved fixing the gels in a solution of 40% ethanol and 10% acetic acid for 1 hour, followed by four washes with deionized water (ddH_2_O) for 20 minutes. The gels were then sensitized with 0.02% sodium thiosulfate for 1 minute, washed three times with ddH_2_O for 20 seconds each, incubated in cold 0.1% silver nitrate solution (0.1% AgNO_3_, 0.02% formaldehyde) for 20 minutes at 4°C, and washed again with ddH_2_O for 20 seconds three times and once for 1 minute. The development was carried out in a solution of 3% sodium carbonate and 0.05% formaldehyde [74]. The stained gel was imaged by ChemiDoc MP Imaging System (Bio-Rad). The samples were resolved on SDS-PAGE and stained with Coomassie Blue for mass spectrometry analysis. After thorough destaining, bands of interest and an equivalent background region from the gel were excised. The excised gel slices were washed twice with 50% acetonitrile in water to remove excess stain. The samples were submitted to the Clinical and Translational Proteomics Service Center at the University of Texas Health Science Center for analysis.

### LC-MS/MS

The gel band samples were subjected to In-gel digestion [75]. An aliquot of the tryptic digest (in 2% acetonitrile/0.1% formic acid in water) was analyzed by LC/MS/MS on an Orbitrap Fusion Tribrid mass spectrometer (ThermoScientific) interfaced with a Dionex UltiMate 3000 Binary RSLCnano System. Peptides were separated onto an analytical C18 column (100μm ID x 25 cm, 5 μm, 18Å Reprosil-Pur C18-AQ beads from Dr Maisch, Ammerbuch-Entringen, Germany) at a flow rate of 350 nl/min. Gradient conditions were: 3%-22% B for 40 min; 22%-35% B for 10min; 35%-90% B for 10 min; 90% B held for 10 min (solvent A, 0.1% formic acid in water; solvent B, 0.1% formic acid in acetonitrile). The peptides were analyzed using a data-dependent acquisition method, Orbitrap Fusion was operated with measurement of FTMS1 at resolutions 120,000 FWHM, scan range 350–1500 m/z, AGC target 2E5, and maximum injection time of 50 ms; During a maximum 3-second cycle time, the ITMS2 spectra were collected at rapid scan rate mode, with HCD NCE 34, 1.6 m/z isolation window, AGC target 1E4, maximum injection time of 35 ms, and dynamic exclusion was employed for 20 seconds.

The raw data files were processed using ThermoScientific Proteome Discoverer software version 1.4, and spectra were searched against the Uniprot- *Mus musculus* (taxonomy id: 10090) or *Schistosoma mansoni* (taxonomy id: 6183) database using Sequest. Trypsin was set as the enzyme with the maximum missed cleavages of 2. The precursor ion tolerance was set to 10 ppm; MS/MS tolerance 0.8 Da. Carbamidomethylation on cysteine residues was used as a static modification; oxidation of methionine, as well as phosphorylation of serine, threonine, and tyrosine, was set as variable modifications. Search results were trimmed to 1% FDR for strict and 5% for relaxed condition using Percolator.

### *In silico* modeling of protein-protein interaction

The amino acid sequence of MEG-8.2 was used to predict its protein structure, with residues 1–20 (N-terminal signal peptide) excluded after identification using PrediSI (http://www.predisi.de/). A 3D model of MEG-8.2 was generated using the Phyre² online server (Protein Homology/analogy Recognition Engine V 2.0) [76]. The generated model was submitted to the YASARA Energy Minimization Server [77] and evaluated using multiple structural validation tools: RAMPAGE (Ramachandran Plot Assessment) [78], ProSA-web (Protein Structure Analysis) [79,80], PROCHECK [81], ERRAT [82], and Verify 3D [83,84]. The CD98hc-LAT1 complex was downloaded from the Protein Data Bank (PDB) [36] under the accession number 6JMQ. Protein-protein docking analyses were performed using the ClusPro online server (https://cluspro.org), which allows primary usage by submitting two files in PDB format [85–87]. All models and protein-protein docking visualizations were carried out using PyMOL (v3.0, The PyMOL Molecular Graphics System, Schrödinger, LLC). For the Cct2–2-MEG 8.2 and MEMO-1-MEG 8.2 models, the structures were generated following the same methodology previously described. The predicted structures of CCT-2 and MEMO-1 were obtained from the AlphaFold protein structure database CCT-2 (https://alphafold.com/entry/P80314), MEMO-1 (https://alphafold.com/entry/Q91VH6). Similarly, the interaction model for Lactoferrin-MEG 8.2 was generated using the same approach, with the predicted structure of Lactoferrin retrieved from the Protein Data Bank (PDB) under accession number 1LFG [88].

### Dot blot assay

Plasma samples were isolated from infected (~7-weeks post-infection) and uninfected Swiss-Webster mice. Circular pieces of PVDF membranes (0.2 µm) were prepared and placed in a 96-well flat-bottom plate. Membranes were first activated with methanol and subsequently washed with deionized water. 50 µL of uninfected and infected plasma samples were added to five separate wells each. 50 µL of 1X PBS was added to a separate well as a blank control. Plates were incubated overnight at 37°C. Following incubation, membranes were washed three times with TBS-T (Tris-buffered saline with Tween-20), blocked for one hour with a blocking buffer, and incubated overnight at 4°C with varying dilutions of the polyclonal anti-MEG-8.2 antibodies we generated (1:2000, 1:1500, 1:1000, 1:500, and 1:100). Following primary antibody incubation, the membranes were washed three times with TBS-T. An HRP-conjugated anti-rabbit secondary antibody (diluted 1:1000) was added and incubated overnight at 4°C. The membranes were washed three times with TBS-T. Detection was performed using DAB (3,3’-diaminobenzidine) substrate solution kit (34002, ThermoScientific) with an incubation period of 5–15 minutes, allowing colorimetric development. The reaction was stopped once the desired signal intensity was achieved, and relative intensity was quantified using ImageJ software.

### Statistical analysis

GraphPad Prism was used to perform appropriate statistical tests. Specific tests are indicated in the figure legends of each figure.

### Oligonucleotides used in this study

Primers used for cloning, RNAi, in situ hybridization, and qPCR are listed in S3 Table.

## Supporting information

S1 Fig Quality control of total RNA used in RNA-seq. (A) Bioanalyzer results of extracted total RNA samples. (B) qPCR of select known EG genes and non-EG genes (*ctsb*) in cDNA synthesized from the extracted RNA samples. The results show specific downregulation of EG genes in *foxA* knockdown. (C) Summary table of RNA concentration/quality and total reads sequenced for each sample.
(TIF)

S2 Fig WISH of genes significantly downregulated in males (A) or females (B). (A) Both genes are significantly downregulated only in males, but show slight enrichment in both males and females. (B) Eggshell protein downregulated in *foxA* RNAi females is not enriched in the EG but is likely enriched in the accessory reproductive tissues (e.g., vitellaria).
(TIF)

S3 Fig FoxA potentially regulates the transcription of EG genes directly but has little effect on other cell types. (A-C) CentriMo analysis of 5kb upstream sequences of 36 EG genes reveals putative forkhead transcription factor binding motifs on most promoters. (A) An overlay of the probability of each motif occurrence. (B) Enriched motifs. (C) A summary table of the location and the significance of each motif. (D) TPM values of cell-type progenitor markers in control and *foxA* RNAi males (left) and females (right). *vwa*, a Mehlis gland marker, shows significantly upregulated (2.6-fold) and downregulated (-1.6-fold) expression levels in males and females, respectively. Two-tailed t-test, ** P < 0.01.
(TIF)

S4 Fig Knockdown efficiencies after RNAi of each candidate gene and their correlation with the feeding phenotype. (A) qPCR of each gene after RNAi-mediated knockdown. (B) Left: 1:1 BLAST between five peptidases downregulated in *foxA* knockdown parasites. Right: qPCR of all five identified peptidases after each gene knockdown. (C) Summary graph categorizing the genes screened based on their knockdown efficiency and leukocyte feeding phenotype. (D) Correlation between the leukocyte feeding phenotype and qPCR measurement of relative *meg-8.2* expression levels in gene knockdowns, showing high variability in the feeding phenotype. (E) Relative fold change of MEG-8 family genes in each MEG-8 family gene knockdown. (F) Relative fold change of non-MEG-8 family genes in *meg-8.1*, *meg-8.3*, and *meg-8.4* knockdown. Error bars in all qPCR graphs indicate the standard deviation of the relative fold change.
(TIF)

S5 Fig *S. mansoni* MEG family alignment. Left: Clustal Omega (v1.2.0); Right: MUSCLE (Algorithm: Neighbor Joining; Distance measure: Jukes-Cantor; Bootstrap: 100 replicates). Sm-MEG-8 family proteins are indicated with red arrows. Amino acid sequences for all of the proteins were derived from the *S. mansoni* genome (V10) available on WormBase ParaSite.
(TIF)

S6 Fig MEG-8 family alignment and a rare case of exon skipping. (A) BLAST of four Sm-MEG-8 proteins across the *Schistosomatidae* family shows orthologs in other species. (B) Sm-MEG-8 alignment. The three predicted helices are indicated by a dotted underline. MEG-8.2 and MEG-8.3 share the most residues (yellow box). Residues shared across all four proteins are marked with a red arrowhead. (C) The regional sequence and location of skipped exon 11, which was found in one out of 10 sequenced clones.
(TIF)

S7 Fig α-MEG-8.2 antibody validation and recombinant expression and purification of MEG-8 proteins and mutants. (A) Representative images of immunofluorescence staining of α-MEG-8.2 antibody after *meg-8.2* and/or *meg-8.3* knockdowns. A single confocal z-section of a male head region is shown for each condition. The number of worms with detectable MEG-8.2 fluorescence is listed in the table. (B) Western blot of α-MEG-8.2 using recombinantly expressed MEG-8 protein lysate. (C) Schematic plasmid map of the bacterial expression vector used to express N-terminal GST-tagged MEG-8 proteins. GST-rMEG-8.2 is shown as an example. (D) SDS-PAGE of GST-only expression and purification steps. (E) Expression and purification of GST-rMEG-8.2. SDS-PAGE (left) shows the highest band corresponding to the expected molecular weight, while a few smaller-sized proteins are observed. Western blot (right) using α-MEG-8.2 antibody confirms that these bands are positively labeled, suggesting that while rMEG-8.2 protein is produced, several degradation products are also in the purified mixture. (F) SDS-PAGE of other members of the Sm-MEG-8 family proteins. (G) SDS-PAGE of rMEG-8.2 truncation mutants. (D – G) Arrowheads indicate the expected band size. L: ladder; + IPTG: IPTG induced; FW: final wash; E1 – 4: elution 1–4.
(TIF)

S8 Fig Cell lytic activity of recombinant MEG-8 proteins and mutants. (A) Hemolysis assay using recombinantly purified MEG-8 proteins. Isolated peripheral blood was treated with indicated proteins for 60 minutes at 37°C. rMEG-8.2 containing α1 region retains the cell lytic activity, as well as rMEG-8.3, but not rMEG-8.1 or rMEG-8.4. EB: elution buffer only. (B) Leukocyte lysis by rMEG-8.2 mutants (top) and other MEG-8 family proteins (bottom). GFP-expressing leukocytes were treated with each protein for 10 minutes at 37°C prior to adding PI and Hoechst33342. The pie chart in the lower-left corner of each image indicates viability. N: total number of cells counted. (C) HeliQuest analysis [89] of each of the predicted helices shows amphipathic properties, with α1 having the highest hydrophobicity and hydrophobic moment. (E) An independent second experiment of the dose curve of the synthetic peptides. EC50 values are largely in agreement with those shown in Fig 4.
(TIF)

S9 Fig MEG-8.3 α3 has similar cytotoxicity as MEG-8.2 α3. (A) Alignment of Sm-MEG-8 family proteins, highlighting the peptides tested. (B) Hemolytic activity of MEG-8.1, MEG-8.3, and MEG-8.4 peptides. (C) Cytotoxic activity of the three lytic peptides, MEG-8.2 α1, MEG-8.2 α3, and MEG-8.3 α3 against host leukocytes. (D) Cytotoxic activity of all MEG-8 family proteins against dissociated parasite cells.
(TIF)

S10 Fig The roles of MEG-8.2 and MEG-8.3 in parasite tissue maintenance. (A) Dissociated parasite cells were treated with GST-only (left) or rMEG-8.2 (right) and labeled with FDA, PI, and Hoechst. Live (FDA + /PI-) cells are marked with arrows, and dead (FDA-/PI+) cells are marked with arrowheads. (B) Schematic of analyzing the EG-to-head area ratio. (C) Dot plots of head area vs EG area (measured via *meg-8.4* (left) or PNA (right)) overlaid with a simple linear regression and its 95% confidence interval. An individual dot represents one adult male. A.U.: arbitrary unit. (D-E) Truncated violin plots showing individual worms’ EG-to-head area ratio with a median and quartiles for different knockdowns. PNA and *meg-8.4* shown in (D) and *meg-32.2* shown in (E). (F) Worm length measurements after the knockdowns. (G) Representative *meg-8.4* FISH images showing a dramatic reduction in the EG size. (H) Histogram showing the percentage of *meg-8.4*-expressing males categorized by the relative expression level. N: Number of adult males analyzed. (I) Representative *meg-32.2* FISH images showing a reduction in EG-to-head area ratio. (J) Number of worms with detectable *meg-32.2* signal. (K) A model describing the potential role of MEG-8.2 and MEG-8.3 in parasite tissue maintenance. Ordinary one-way ANOVA followed by Tukey’s multiple comparison tests were performed for (D), (E), and (F). * P < 0.05; ** P < 0.01; *** P < 0.001; **** P < 0.0001.
(TIF)

S11 Fig Modeling interaction between LTF and MEG-8.2. (A) MEG-8.2 residues with an interaction distance <4Å are highlighted in magenta. (B) Dot blot of MEG-8.2 in plasma lysate derived from infected and uninfected mice using a range of dilutions of anti-MEG-8.2 antibodies.
(TIF)

S1 Table Differential expression analysis of *foxA* RNAi RNA-seq. (XLSX)

S2 Table LC-MS/MS analysis of rMEG-8.2 pull-down hits. (XLSX)

S3 Table List of oligonucleotides and peptides used in this study. (XLSX)

S4 Table Plasmid sequences for all constructs used for recombinant protein expression. (XLSX)
