# Supplementary figures and images for: Schistosome esophageal gland factor MEG-8.2 drives host cell lysis and interacts with host immune proteins
Source: PLoS Pathog. 2026 Mar 11;22(3):e1014044. doi: 10.1371/journal.ppat.1014044 (PMC12998952; doi:10.1371/journal.ppat.1014044)

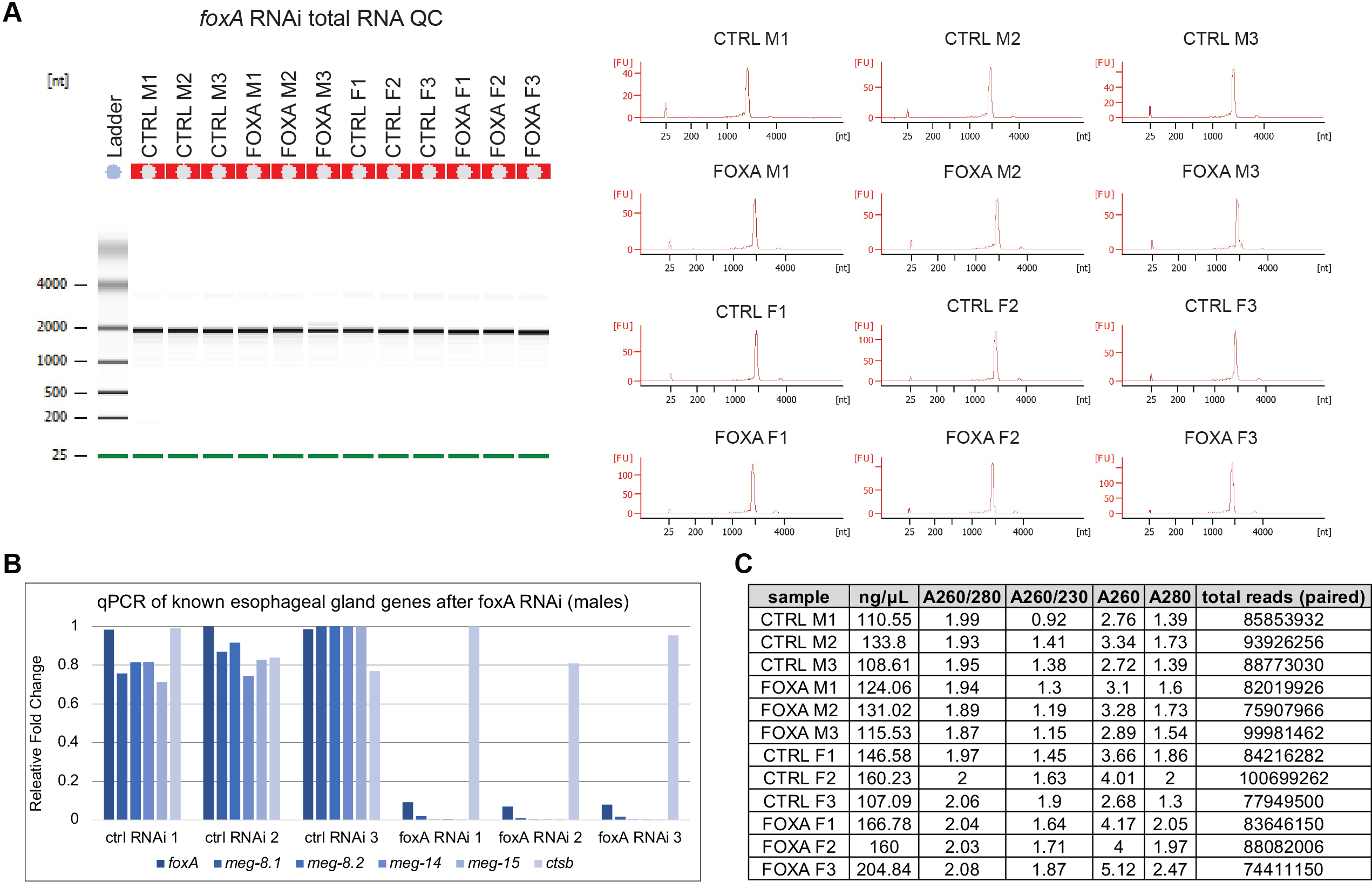

Supplement: S1 Fig — (A) Bioanalyzer results of extracted total RNA samples. (B) qPCR of select known EG genes and non-EG genes (ctsb) in cDNA synthesized from the extracted RNA samples. The results show specific downregulation of EG genes in foxA knockdown. (C) Summary table of RNA concentration/quality and total reads sequenced for each sample. (TIF) [file ppat.1014044.s001.tif]

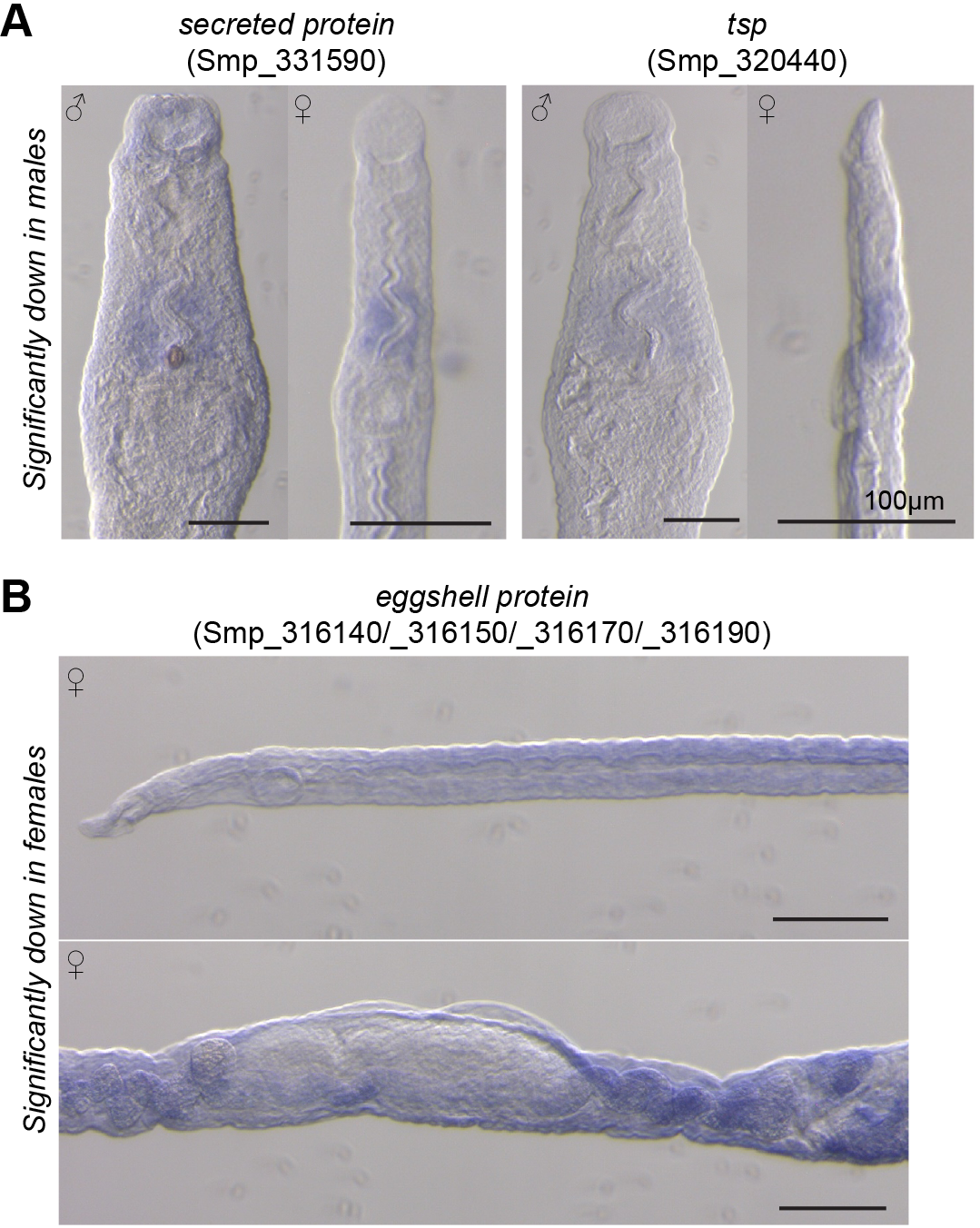

Supplement: S2 Fig — (A) Both genes are significantly downregulated only in males, but show slight enrichment in both males and females. (B) Eggshell protein downregulated in foxA RNAi females is not enriched in the EG but is likely enriched in the accessory reproductive tissues (e.g., vitellaria). (TIF) [file ppat.1014044.s002.tif]

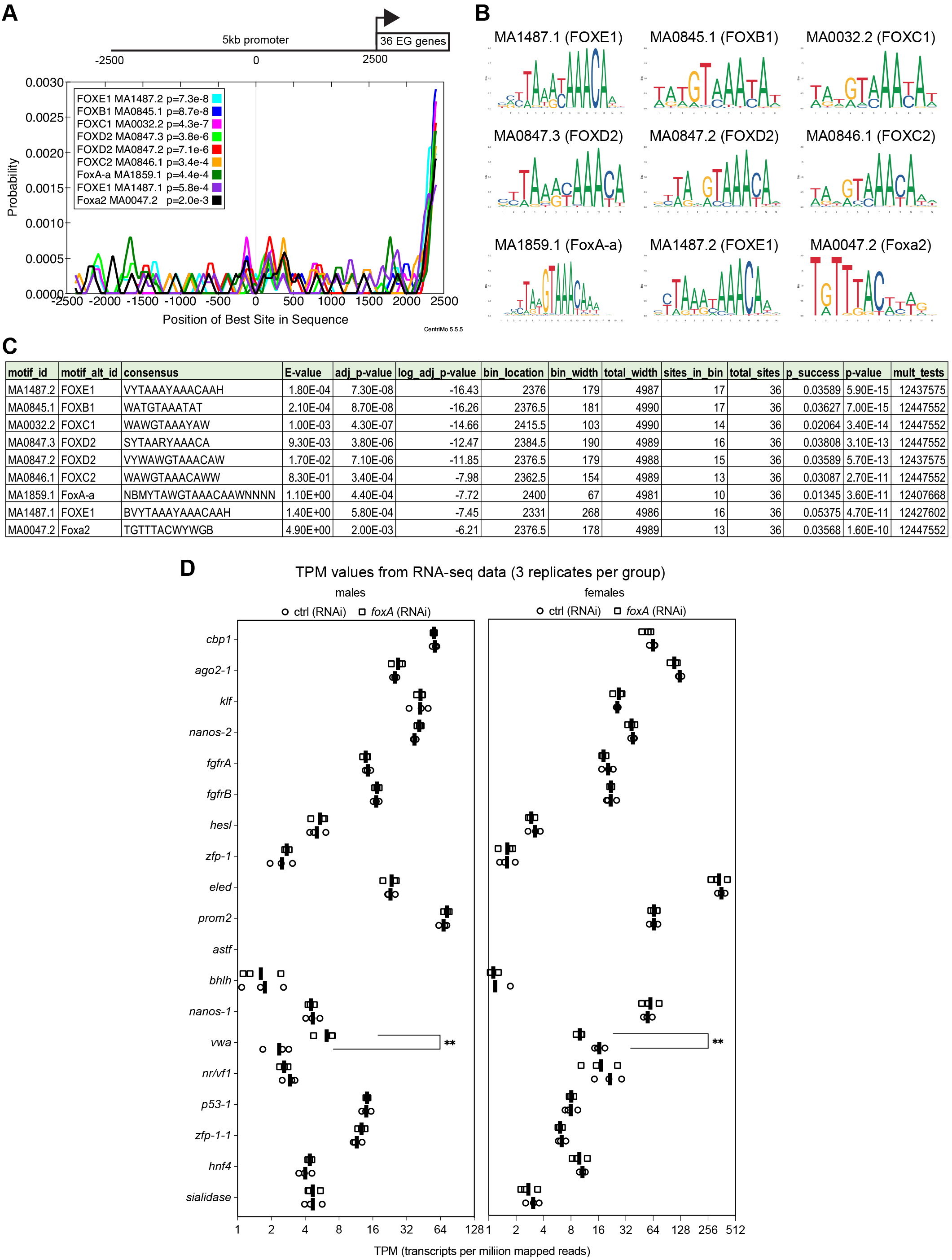

Supplement: S3 Fig — (A-C) CentriMo analysis of 5kb upstream sequences of 36 EG genes reveals putative forkhead transcription factor binding motifs on most promoters. (A) An overlay of the probability of each motif occurrence. (B) Enriched motifs. (C) A summary table of the location and the significance of each motif. (D) TPM values of cell-type progenitor markers in control and foxA RNAi males (left) and females (right). vwa, a Mehlis gland marker, shows significantly upregulated (2.6-fold) and downregulated (-1.6-fold) expression levels in males and females, respectively. Two-tailed t-test, ** P < 0.01. (TIF) [file ppat.1014044.s003.tif]

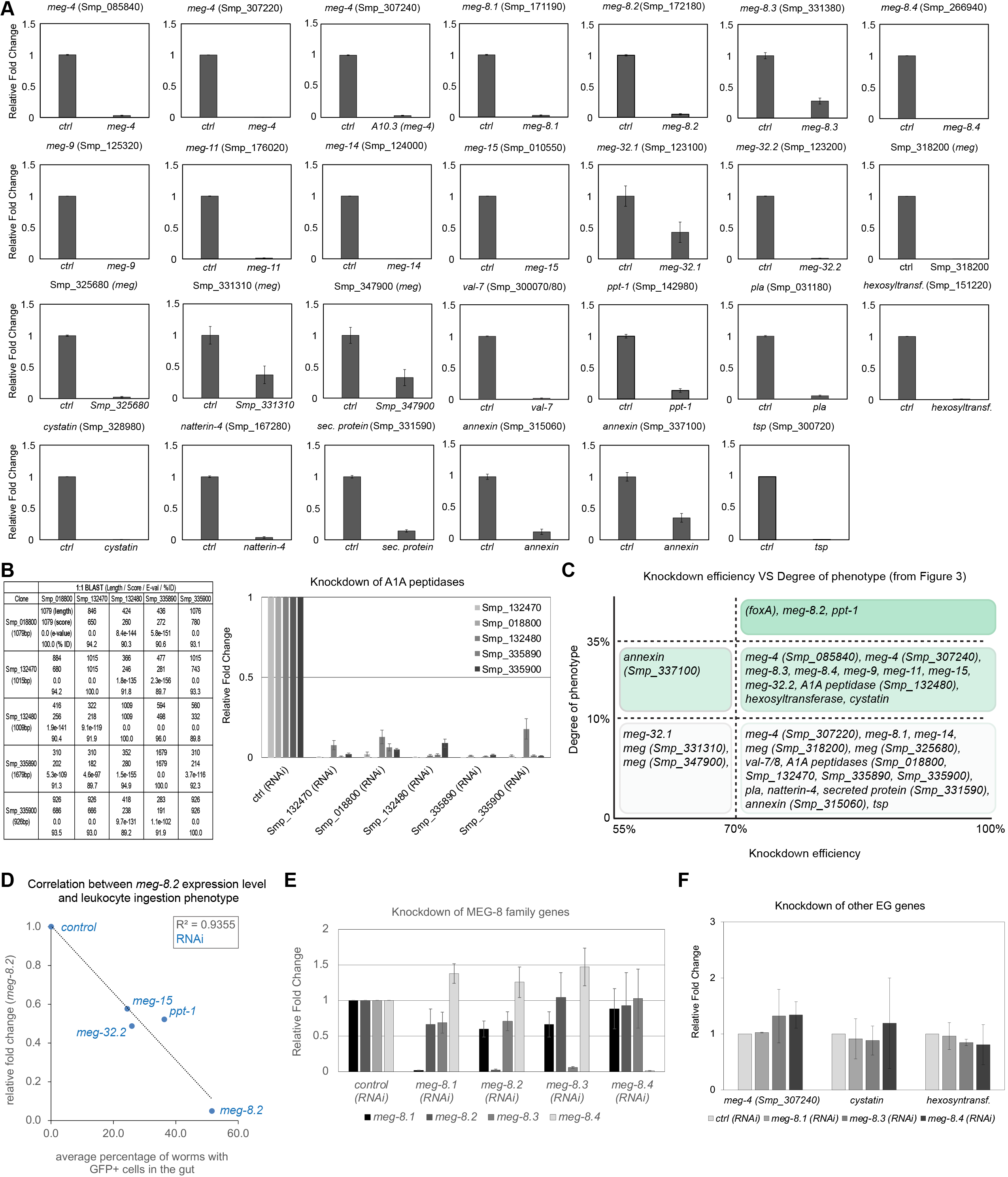

Supplement: S4 Fig — (A) qPCR of each gene after RNAi-mediated knockdown. (B) Left: 1:1 BLAST between five peptidases downregulated in foxA knockdown parasites. Right: qPCR of all five identified peptidases after each gene knockdown. (C) Summary graph categorizing the genes screened based on their knockdown efficiency and leukocyte feeding phenotype. (D) Correlation between the leukocyte feeding phenotype and qPCR measurement of relative meg-8.2 expression levels in gene knockdowns, showing high variability in the feeding phenotype. (E) Relative fold change of MEG-8 family genes in each MEG-8 family gene knockdown. (F) Relative fold change of non-MEG-8 family genes in meg-8.1, meg-8.3, and meg-8.4 knockdown. Error bars in all qPCR graphs indicate the standard deviation of the relative fold change. (TIF) [file ppat.1014044.s004.tif]

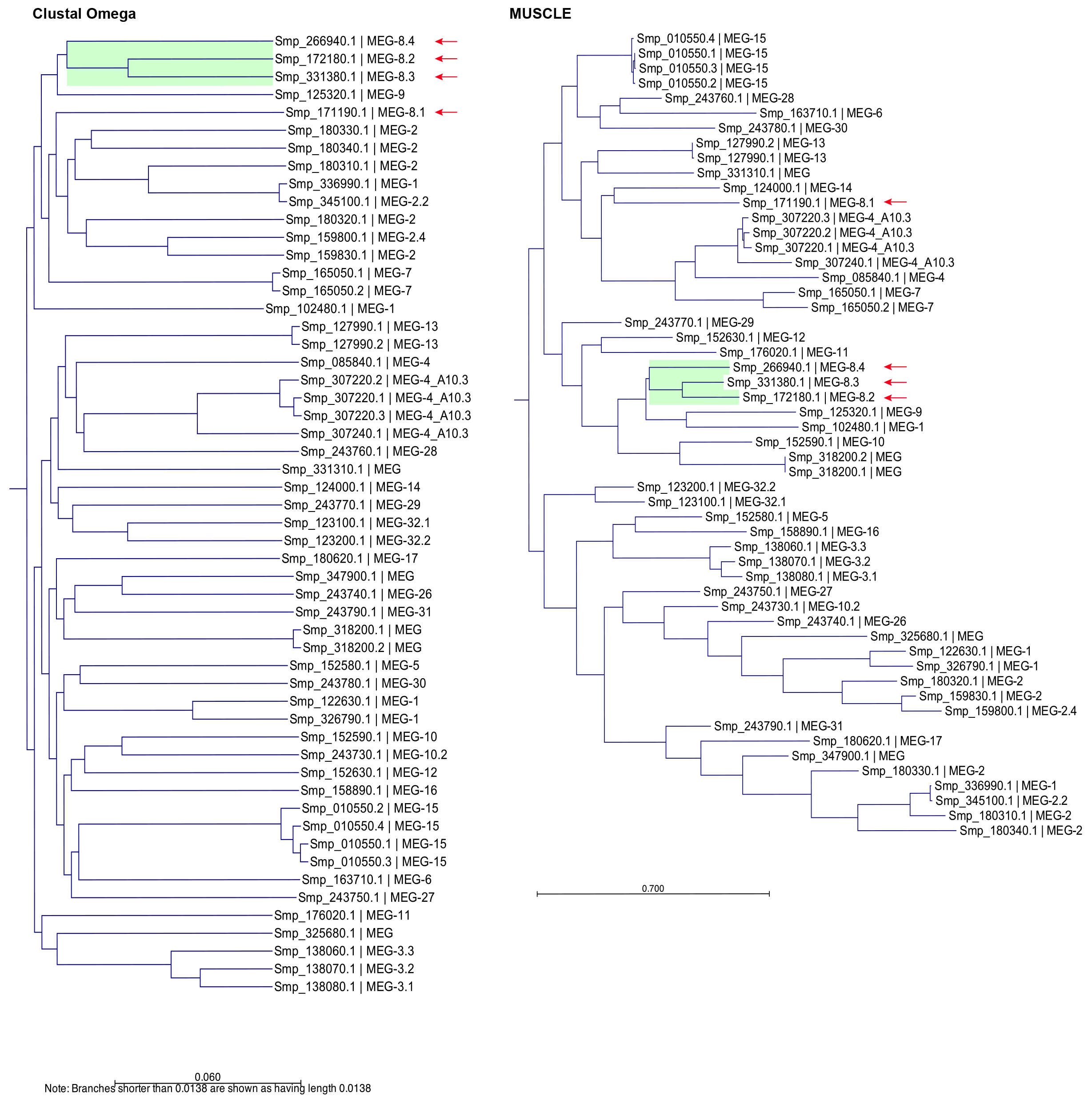

Supplement: S5 Fig — Left: Clustal Omega (v1.2.0); Right: MUSCLE (Algorithm: Neighbor Joining; Distance measure: Jukes-Cantor; Bootstrap: 100 replicates). Sm-MEG-8 family proteins are indicated with red arrows. Amino acid sequences for all of the proteins were derived from the S. mansoni genome (V10) available on WormBase ParaSite. (TIF) [file ppat.1014044.s005.tif]

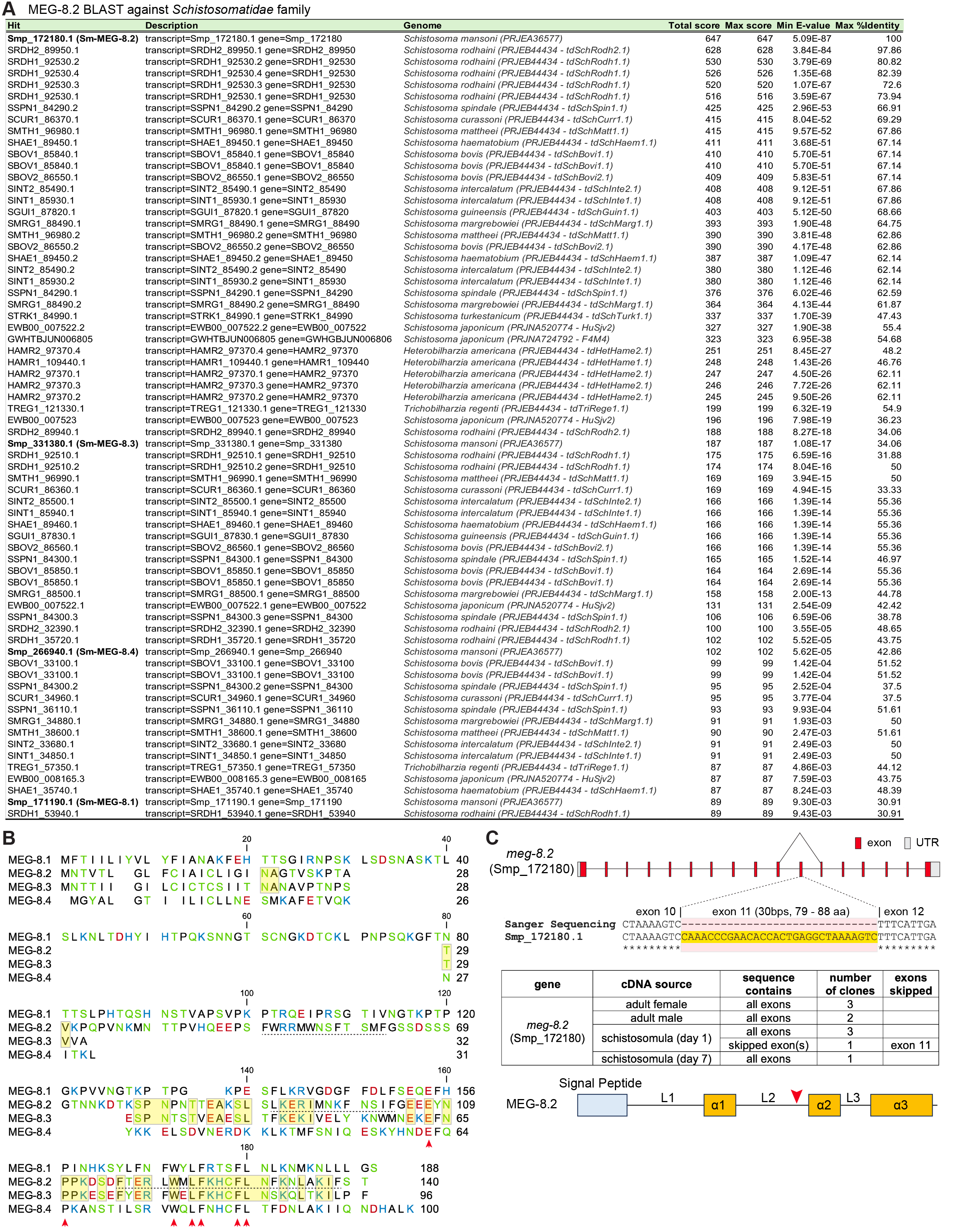

Supplement: S6 Fig — (A) BLAST of four Sm-MEG-8 proteins across the Schistosomatidae family shows orthologs in other species. (B) Sm-MEG-8 alignment. The three predicted helices are indicated by a dotted underline. MEG-8.2 and MEG-8.3 share the most residues (yellow box). Residues shared across all four proteins are marked with a red arrowhead. (C) The regional sequence and location of skipped exon 11, which was found in one out of 10 sequenced clones. (TIF) [file ppat.1014044.s006.tif]

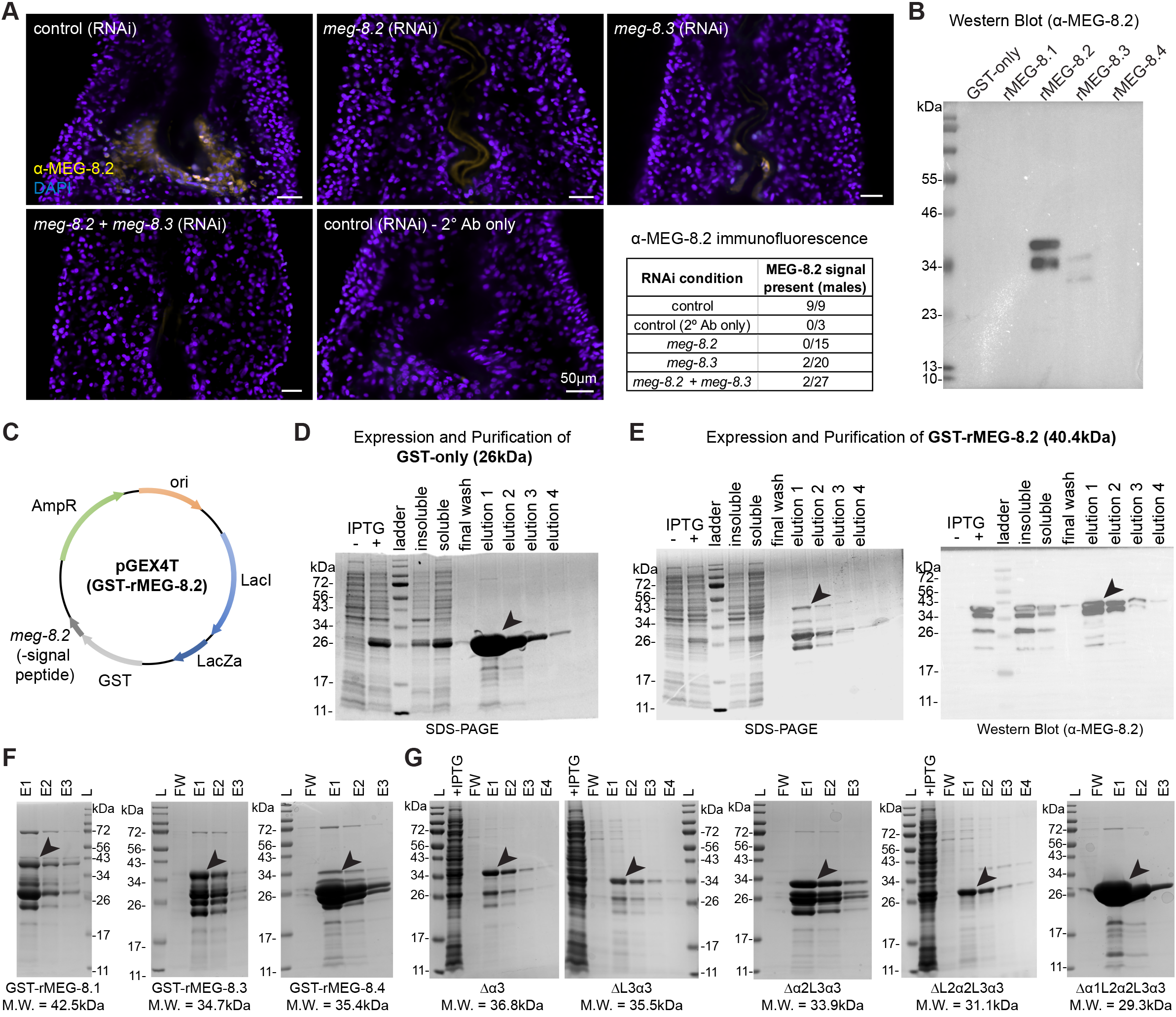

Supplement: S7 Fig — (A) Representative images of immunofluorescence staining of α-MEG-8.2 antibody after meg-8.2 and/or meg-8.3 knockdowns. A single confocal z-section of a male head region is shown for each condition. The number of worms with detectable MEG-8.2 fluorescence is listed in the table. (B) Western blot of α-MEG-8.2 using recombinantly expressed MEG-8 protein lysate. (C) Schematic plasmid map of the bacterial expression vector used to express N-terminal GST-tagged MEG-8 proteins. GST-rMEG-8.2 is shown as an example. (D) SDS-PAGE of GST-only expression and purification steps. (E) Expression and purification of GST-rMEG-8.2. SDS-PAGE (left) shows the highest band corresponding to the expected molecular weight, while a few smaller-sized proteins are observed. Western blot (right) using α-MEG-8.2 antibody confirms that these bands are positively labeled, suggesting that while rMEG-8.2 protein is produced, several degradation products are also in the purified mixture. (F) SDS-PAGE of other members of the Sm-MEG-8 family proteins. (G) SDS-PAGE of rMEG-8.2 truncation mutants. (D – G) Arrowheads indicate the expected band size. L: ladder; + IPTG: IPTG induced; FW: final wash; E1 – 4: elution 1–4. (TIF) [file ppat.1014044.s007.tif]

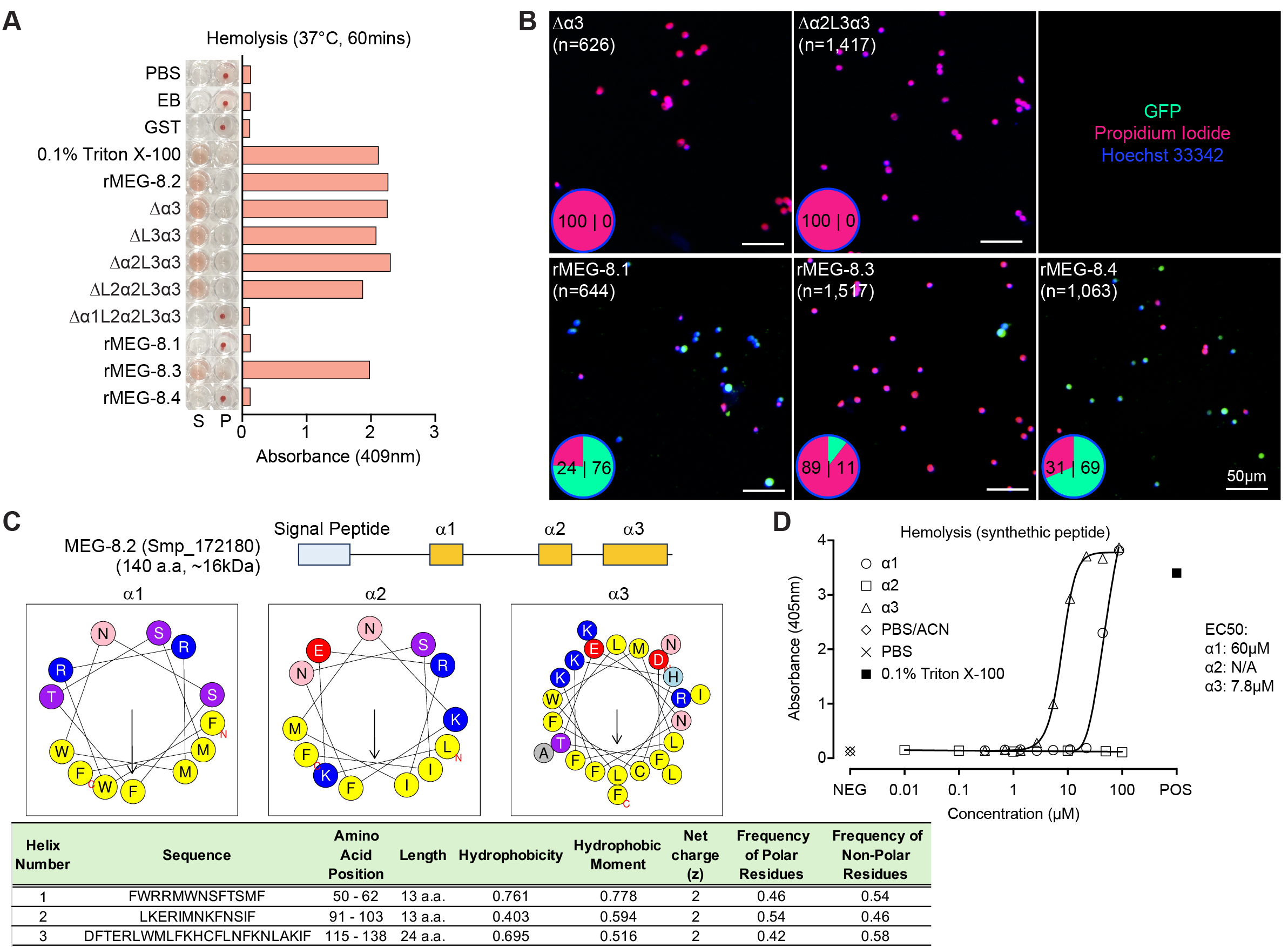

Supplement: S8 Fig — (A) Hemolysis assay using recombinantly purified MEG-8 proteins. Isolated peripheral blood was treated with indicated proteins for 60 minutes at 37°C. rMEG-8.2 containing α1 region retains the cell lytic activity, as well as rMEG-8.3, but not rMEG-8.1 or rMEG-8.4. EB: elution buffer only. (B) Leukocyte lysis by rMEG-8.2 mutants (top) and other MEG-8 family proteins (bottom). GFP-expressing leukocytes were treated with each protein for 10 minutes at 37°C prior to adding PI and Hoechst33342. The pie chart in the lower-left corner of each image indicates viability. N: total number of cells counted. (C) HeliQuest analysis [89] of each of the predicted helices shows amphipathic properties, with α1 having the highest hydrophobicity and hydrophobic moment. (E) An independent second experiment of the dose curve of the synthetic peptides. EC50 values are largely in agreement with those shown in Fig 4. (TIF) [file ppat.1014044.s008.tif]

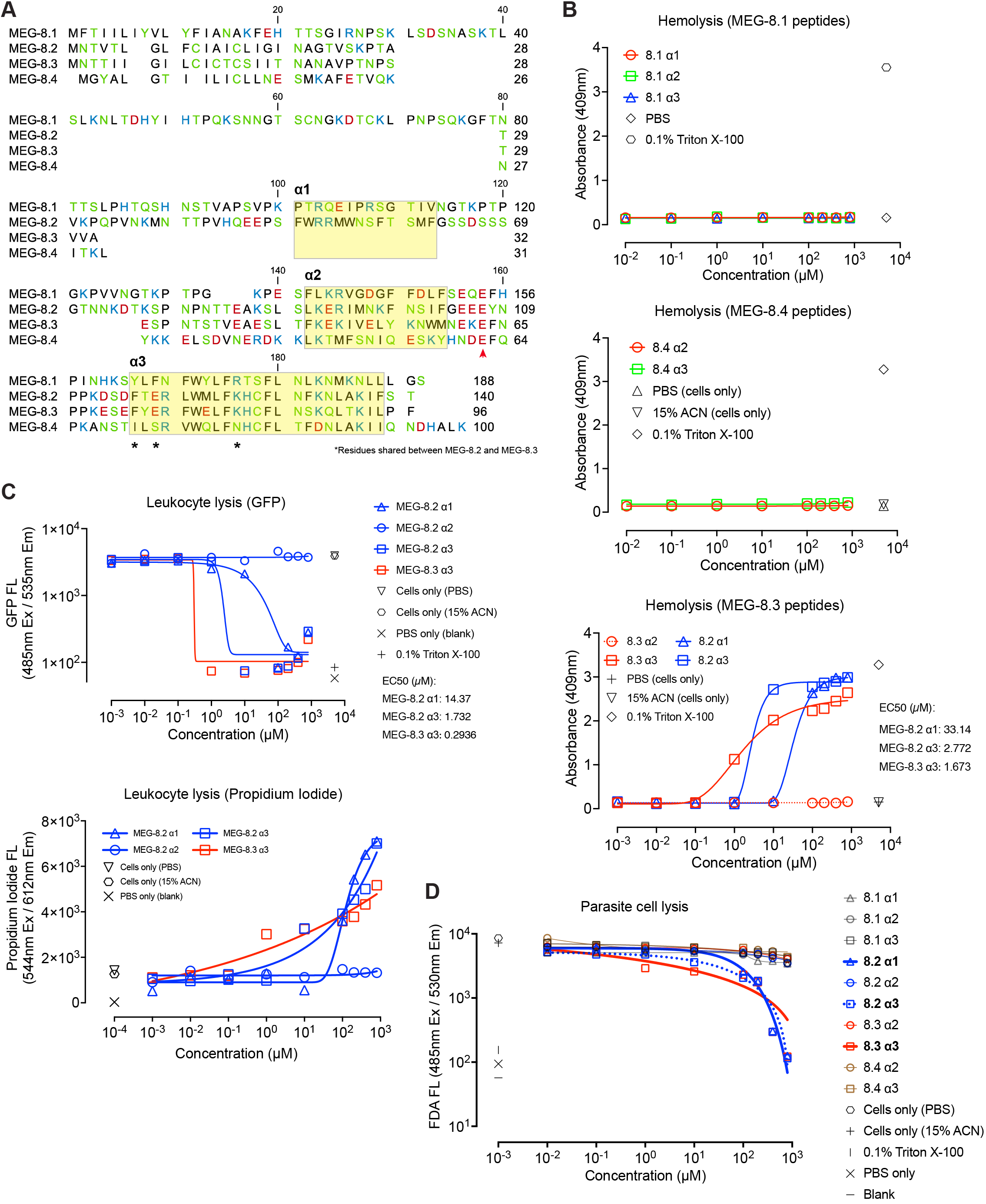

Supplement: S9 Fig — (A) Alignment of Sm-MEG-8 family proteins, highlighting the peptides tested. (B) Hemolytic activity of MEG-8.1, MEG-8.3, and MEG-8.4 peptides. (C) Cytotoxic activity of the three lytic peptides, MEG-8.2 α1, MEG-8.2 α3, and MEG-8.3 α3 against host leukocytes. (D) Cytotoxic activity of all MEG-8 family proteins against dissociated parasite cells. (TIF) [file ppat.1014044.s009.tif]

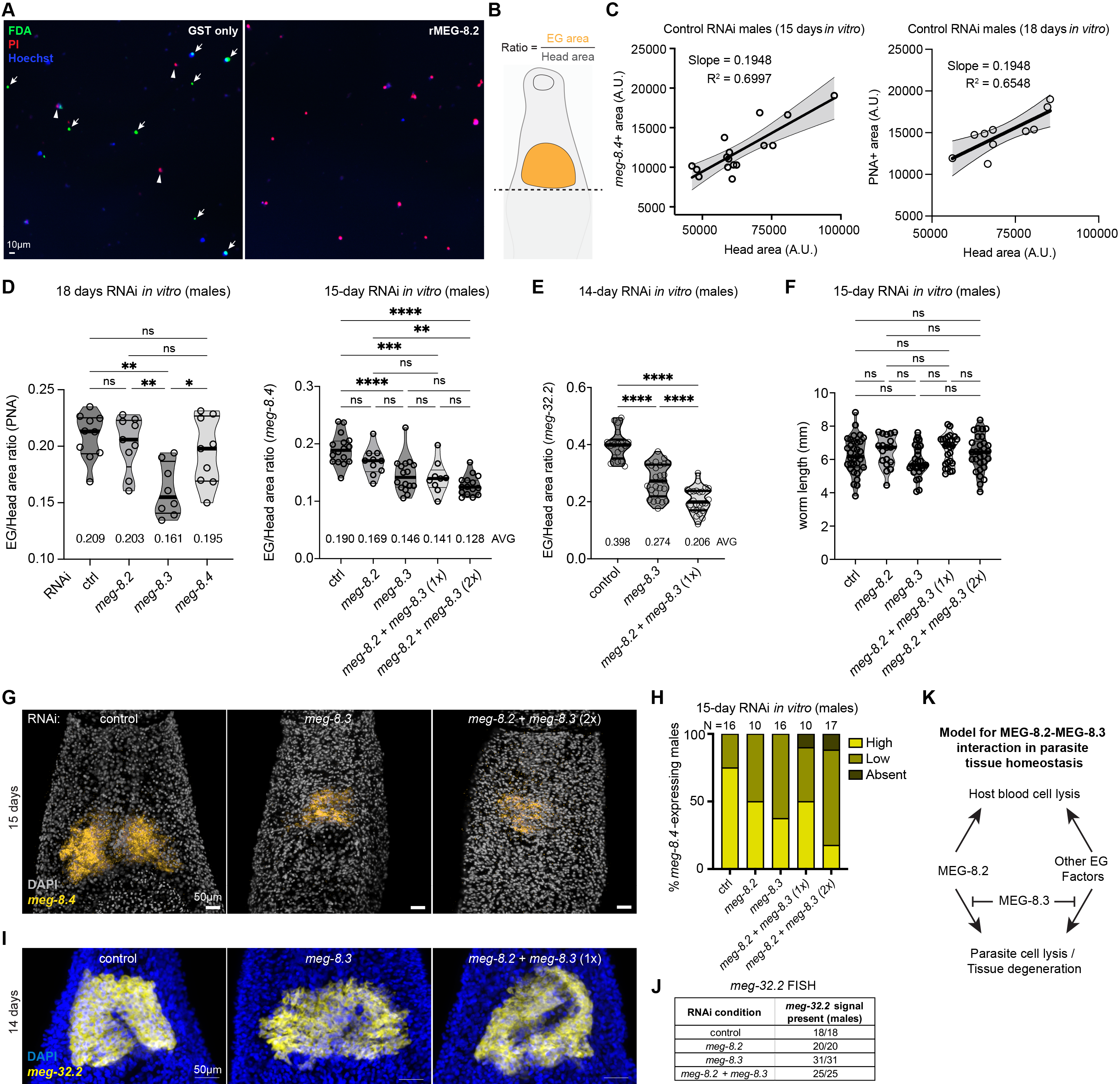

Supplement: S10 Fig — (A) Dissociated parasite cells were treated with GST-only (left) or rMEG-8.2 (right) and labeled with FDA, PI, and Hoechst. Live (FDA + /PI-) cells are marked with arrows, and dead (FDA-/PI+) cells are marked with arrowheads. (B) Schematic of analyzing the EG-to-head area ratio. (C) Dot plots of head area vs EG area (measured via meg-8.4 (left) or PNA (right)) overlaid with a simple linear regression and its 95% confidence interval. An individual dot represents one adult male. A.U.: arbitrary unit. (D-E) Truncated violin plots showing individual worms’ EG-to-head area ratio with a median and quartiles for different knockdowns. PNA and meg-8.4 shown in (D) and meg-32.2 shown in (E). (F) Worm length measurements after the knockdowns. (G) Representative meg-8.4 FISH images showing a dramatic reduction in the EG size. (H) Histogram showing the percentage of meg-8.4-expressing males categorized by the relative expression level. N: Number of adult males analyzed. (I) Representative meg-32.2 FISH images showing a reduction in EG-to-head area ratio. (J) Number of worms with detectable meg-32.2 signal. (K) A model describing the potential role of MEG-8.2 and MEG-8.3 in parasite tissue maintenance. Ordinary one-way ANOVA followed by Tukey’s multiple comparison tests were performed for (D), (E), and (F). * P < 0.05; ** P < 0.01; *** P < 0.001; **** P < 0.0001. (TIF) [file ppat.1014044.s010.tif]

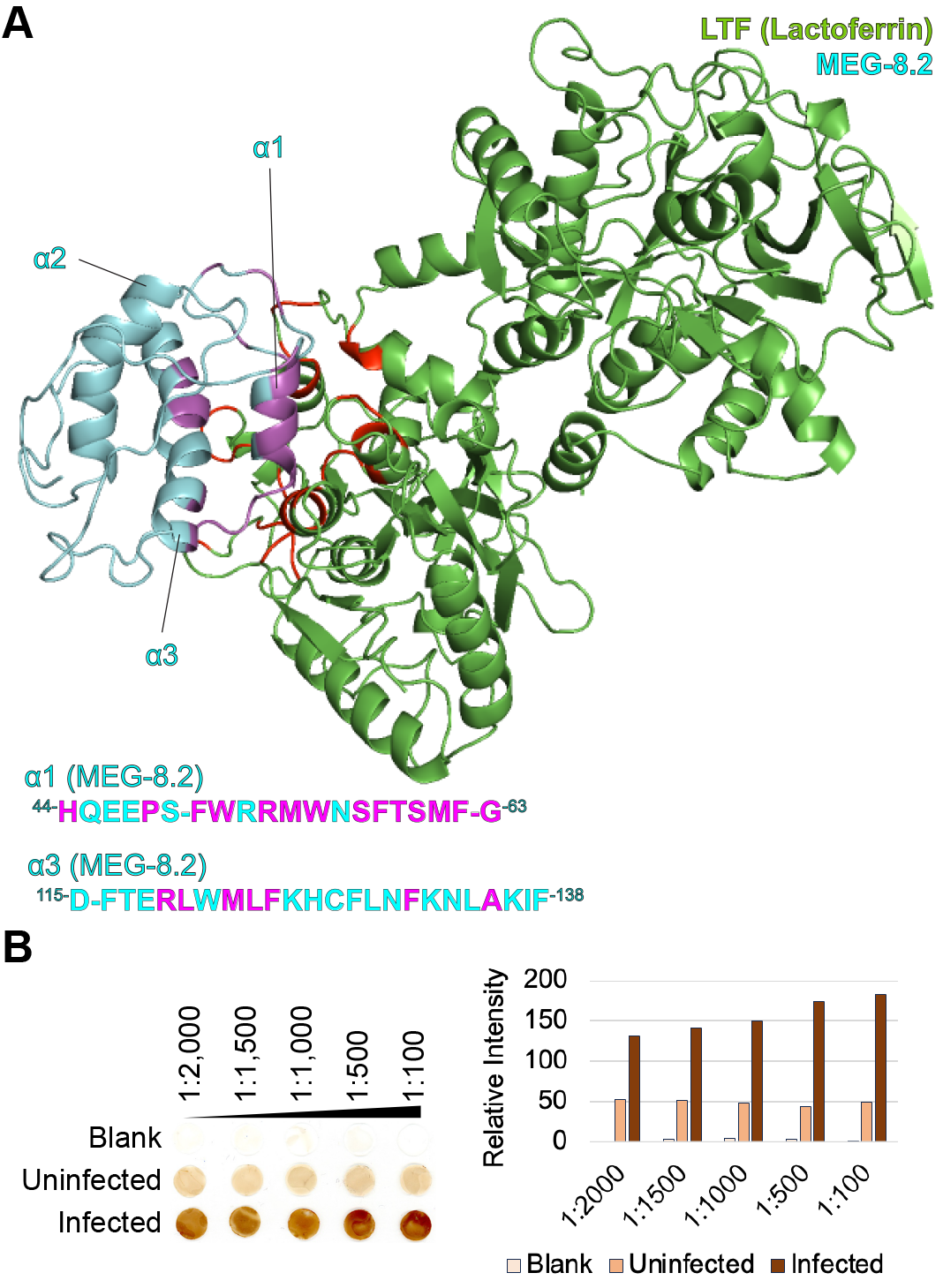

Supplement: S11 Fig — (A) MEG-8.2 residues with an interaction distance <4Å are highlighted in magenta. (B) Dot blot of MEG-8.2 in plasma lysate derived from infected and uninfected mice using a range of dilutions of anti-MEG-8.2 antibodies. (TIF) [file ppat.1014044.s011.tif]
